# Author Correction: Structural basis of neuropeptide Y signaling through Y1 receptor

**DOI:** 10.1038/s41467-022-28837-0

**Published:** 2022-02-25

**Authors:** Chaehee Park, Jinuk Kim, Seung-Bum Ko, Yeol Kyo Choi, Hyeongseop Jeong, Hyeonuk Woo, Hyunook Kang, Injin Bang, Sang Ah Kim, Tae-Young Yoon, Chaok Seok, Wonpil Im, Hee-Jung Choi

**Affiliations:** 1 Department of Biological Sciences, Seoul National University, Seoul, 08826 Republic of Korea; 2 Departments of Biological Sciences and Chemistry, Lehigh University, Bethlehem, PA 18015 USA; 3 Center for Electron Microscopy Research, Korea Basic Science Institute, Chungcheongbuk-do, 28119 Republic of Korea; 4 Department of Chemistry, Seoul National University, Seoul, 08826 Republic of Korea; 5 Institute for Molecular Biology and Genetics, Seoul National University, Seoul, 08826 Republic of Korea; 6 Present Address: Perlmutter Cancer Center, New York University Langone Health, and Department of Biochemistry and Molecular Pharmacology, New York University School of Medicine, New York, NY 10016 USA

**Keywords:** Cryoelectron microscopy, G protein-coupled receptors, Neurochemistry

Correction to: *Nature Communications* 10.1038/s41467-022-28510-6, published online 14 February 2022.

In this article, the affiliation Institute for Molecular Biology and Genetics, Seoul National University, Seoul 08826, Republic of Korea for Tae-Young Yoon was missing. The original article has been corrected.

