# Supplementary material for: Structural basis of neuropeptide Y signaling through Y1 receptor
Source: Nat Commun. 2022 Feb 14;13:853. doi: 10.1038/s41467-022-28510-6 (PMC8844075; doi:10.1038/s41467-022-28510-6)
Supplement: Supplementary file 1 — Supplementary Information [file 41467_2022_28510_MOESM1_ESM.pdf]

Structural basis of neuropeptide Y signaling through Y1 receptor

Supplementary Tables 1 and 2
Supplementary Figures 1–29
References

Supplementary Table 1 Cryo-EM data collection, refinement and validation statistics.

	NPY–Y_1R–$G\alpha_{i1}\beta\gamma$–scFv16 (EMD-31979) (PDB 7VGX)
**Data collection and processing**	
Magnification	75,000
Voltage (kV)	300
Electron exposure (e–/Å^2)	40
Defocus range (μm)	−1.25 to −2.75
Pixel size (Å)	0.865
Symmetry imposed	C1
Initial particle images (no.)	1,339,224
Final particle images (no.)	233,117
Map resolution (Å)	3.2
FSC threshold	0.143
Map resolution range (Å)	n/a
**Refinement**	
Initial model used (PDB code)	5ZBH, 6OS9
Model resolution (Å)	3.3
FSC threshold	0.5
Model resolution range (Å)	n/a
Map sharpening *B* factor (Å^2)	-147.1
Model composition	
Non-hydrogen atoms	9,044
Protein residues	1,148
Ligands	TYC: 1
B factors (Å^2)	
Protein	71.51
Ligand	97.41
R.m.s. deviations	
Bond lengths (Å)	0.004
Bond angles (°)	0.655
Validation	
MolProbity[1] score	1.50
Clashscore	5.79
Poor rotamers (%)	0.00
Ramachandran plot	
Favored (%)	96.89
Allowed (%)	3.11
Disallowed (%)	0.00
EMRinger[2] score	3.49

Supplementary Table 2 Ca^{2+} signaling assays of wild-type and mutant Y_1 receptors with different peptide ligands.

a

Y_1R Mutants	NPY (1–36)		
	EC_{50} (nM) (pEC_{50} ± SEM)	n*	X-fold over WT†
WT	4.7 (8.3 ± 0.061)	17	1
$Q120^{3.32}A$	8.7 (8.1 ± 0.18)	3	1.9
$Q219^{5.46}L$	19 (7.7 ± 0.19)	3	4.0
$N283^{6.55}A$	400 (6.4 ± 0.26)	3	85
$F286^{6.58}V$	160 (6.8 ± 0.83)	4	34
$F184^{ECL2}A$	180 (6.7 ± 0.25)	3	38
$F199^{ECL2}A$	11 (8.0 ± 0.24)	3	2.3
$F202^{ECL2}A$	37 (7.4 ± 0.31)	3	7.9
$R208^{5.35}A$	8.6 (8.1 ± 0.23)	3	1.8
Y_1RΔ25	1.5 (8.8 ± 0.24)	3	0.32
Y_1RΔ31	11 (8.0 ± 0.22)	3	2.3
$L26^{N}A/F28^{N}A$	13 (7.9 ± 0.40)	3	2.8

b

Neuropeptide Y	EC_{50} (nM) (pEC_{50} ± SEM)	n	X-fold over WT†
1–36	4.7 (8.3 ± 0.061)	17	1
3–36	86 (7.1 ± 0.30)	3	18
18–36	1400 (5.9 ± 0.15)	3	300
AA–36	170 (6.8 ± 0.29)	3	36

c

Peptide YY	EC_{50} (nM) (pEC_{50} ± SEM)	n	X-fold over WT†
1–36	6.1 (8.2 ± 0.28)	5	1
3–36	77 (7.1 ± 0.52)	5	13

d

Pancreatic Polypeptide	EC_{50} (nM) (pEC_{50} ± SEM)	N	X-fold over NPY (1–36)
1–36	300 (6.5 ± 0.33)	4	64

Supplementary Table 2 a EC_{50} (pEC_{50} ± SEM) values of wild-type (WT) and various Y_1R mutants measured by Ca^{2+} assays are shown. The dose response curves for each mutant are shown in **Supplementary Figures 11**, **17**, and **22**. **b-d** EC_{50} (pEC_{50} ± SEM) values of wild-type Y_1R upon treatment with truncated NPY (3–36, 18–36), mutant NPY (1–36 with Y1A/P2A mutations, abbreviated as AA-36), PYY (1–36, 3–36), and PP (1–36) are shown. The dose response curves are shown in **Figure 5b** and **Supplementary Figure 26**.

*Sample size; number of independent experiments
† The X-fold over WT; the ratio of **a** EC_{50} (Y_1R mutant)/EC_{50} (Y_1R WT), **b** EC_{50} (NPY analogue)/EC_{50} (NPY), **c** EC_{50} (truncated PYY)/EC_{50} (PYY)

Supplementary Figure 1 Subtype-specific G protein recruitment of Y_1R by NPY treatment. Bioluminescence Resonance Energy Transfer (BRET) assays showed that Y_1R recruits only G_i upon NPY treatment. Bar and error bar for each sample represent the mean and S.E.M. (standard error of mean) of n=six independent experiments, respectively. Each circle represents an individual data point from the experiment. Source data are provided as a Source Data file.

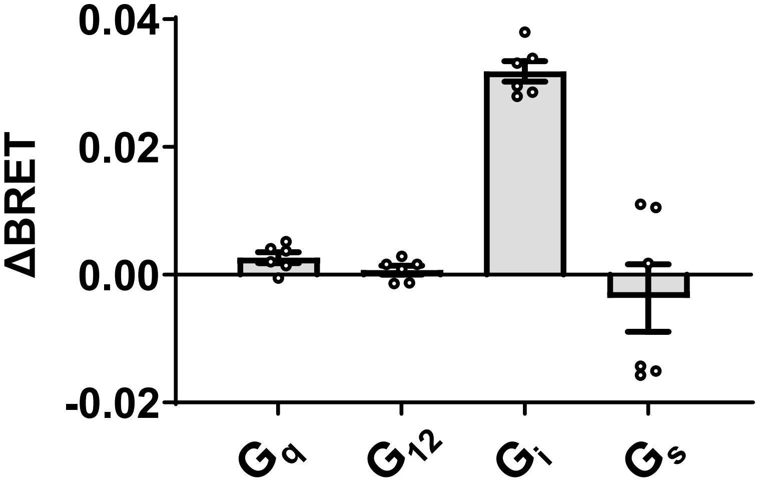

Supplementary Figure 2 Purification of the NPY–Y_1R–G_{i1}–scFv16 complex. a Size exclusion chromatography profile of the NPY–Y_1R–G_{i1}–scFv16 complex. Eluted fractions indicated by beige shaded area were pooled. Source data are provided as a Source Data file. **b** SDS-PAGE analysis of the purified NPY–Y_1R–G_{i1}–scFv16 complex. Gγ_2 and NPY peptide are not shown in this gel due to their small molecular weights (MW). Four independent experiments of complex purification were carried out, which consistently yielded protein purities similar to that shown on this SDS-PAGE gel. Uncropped gel image is provided at the end of the Supplementary Information file and provided as a Source Data file.

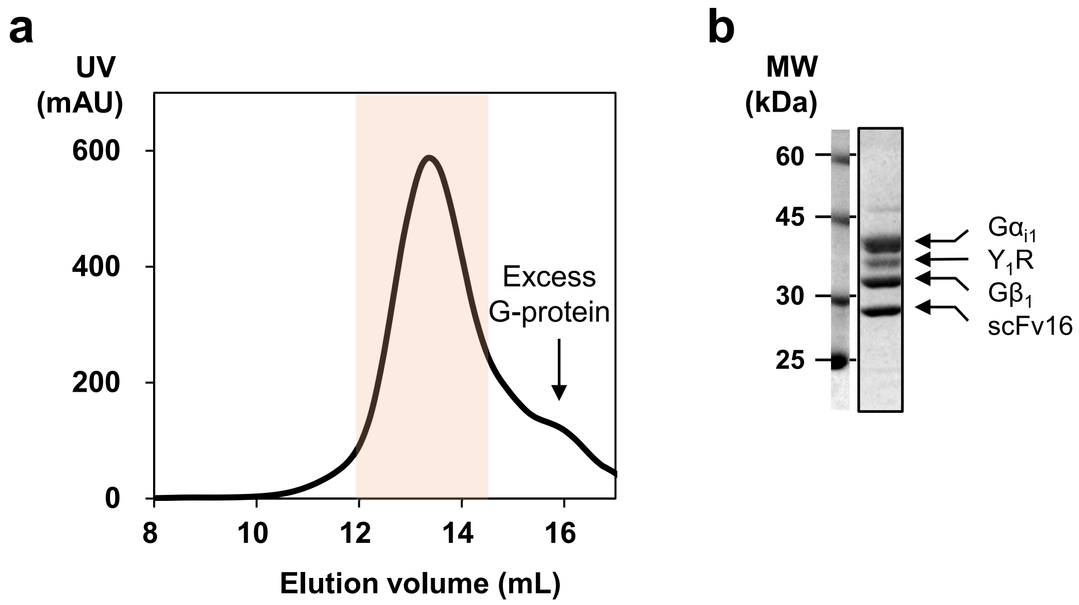

Supplementary Figure 3 Cryo-EM workflows for structure determination of the NPY–Y_1R–G_{i1} complex. a Representative cryo-EM micrograph (scale bar, 30 nm) and 2D classification averages (scale bar, 5 nm). **b** Cryo-EM data processing flowcharts. **c** The fourier shell correlation (FSC) curve of globally refined NPY–Y_1R–G_{i1}–scFv16 complex, locally refined G_{i1}–scFv16 complex and NPY–Y_1R. **d** The Euler angle distribution of particles used in the final refinement. **e** Local resolution estimation of each refined cryo-EM map.

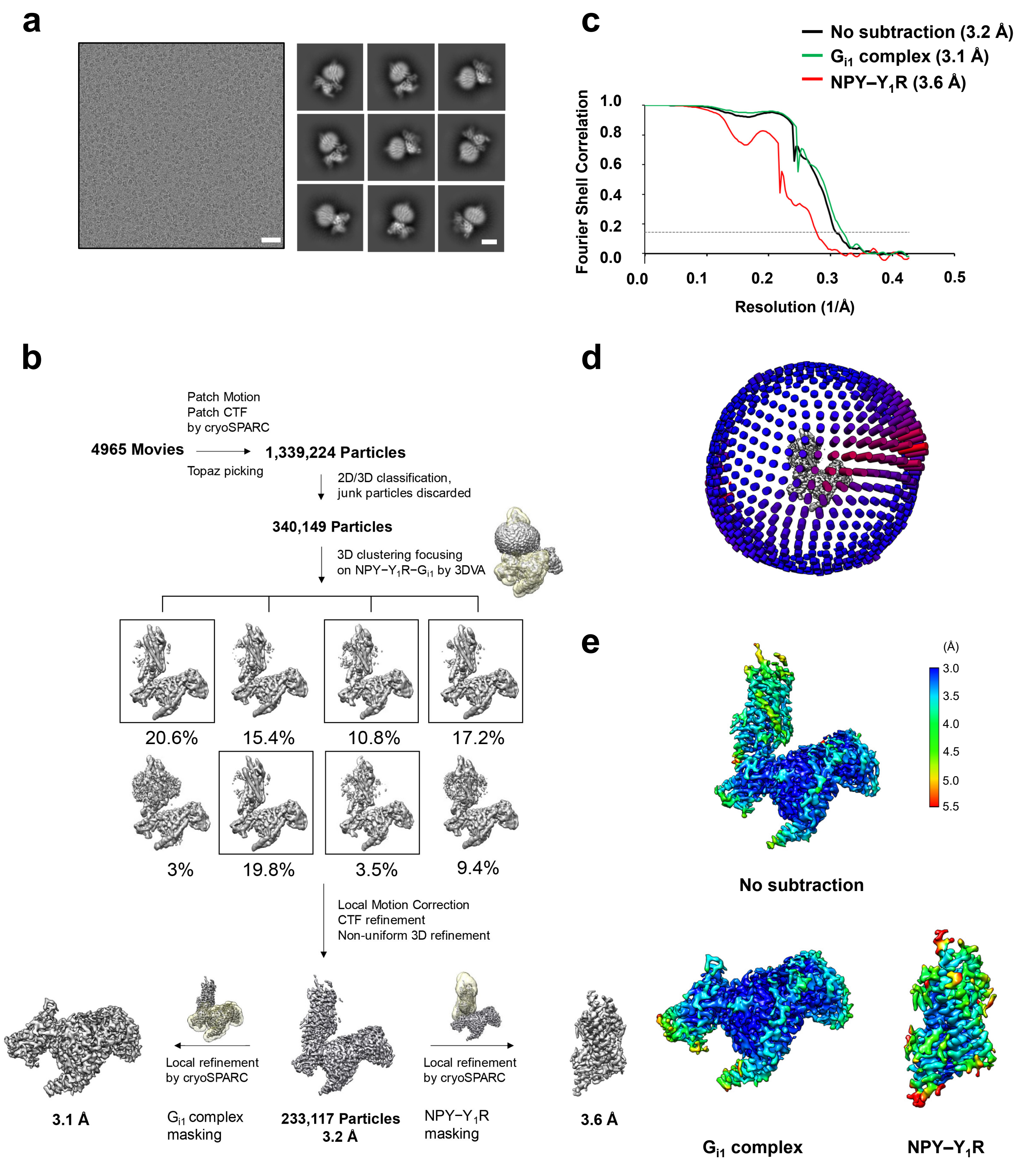

Supplementary Figure 4 Cryo-EM density representation. Cryo-EM density map and model are shown for **a** seven transmembrane helices of Y$_1$R, NPY (NPY–Y$_1$R focused map), αN and α5 of Gα$_{i1}$, parts of Gβ$_1$ and Gγ$_2$ (G$_{i1}$ complex focused map), and **b** (D/E)R(Y/H) motif, NPxxY motif and the connector region.

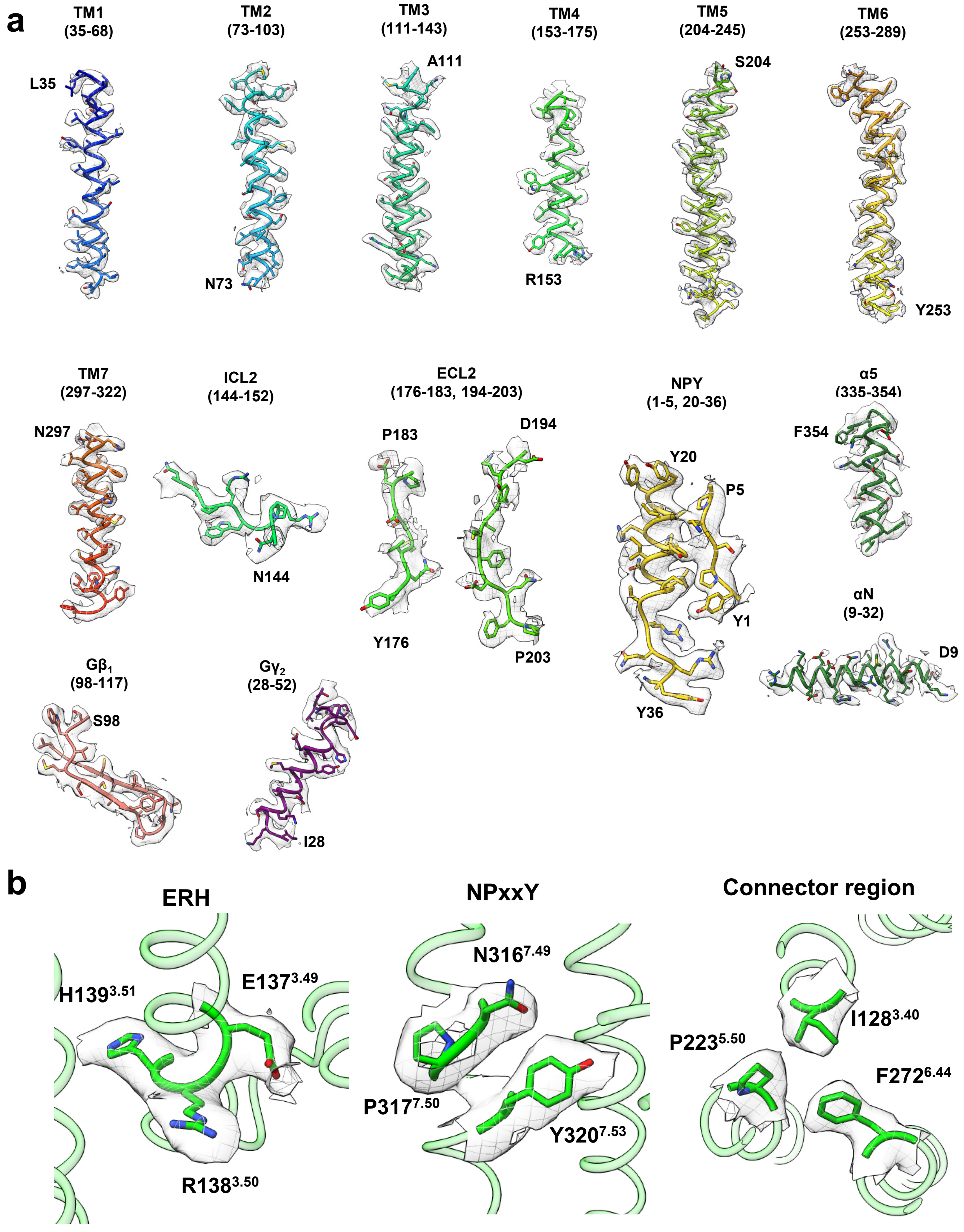

Supplementary Figure 5 Solvent accessible ligand binding volume. The antagonist-bound (light grey: 5ZBQ, grey: 5ZBH) and NPY-bound (green) structures of Y_1R are shown with the volumes of solvent accessible ligand binding cavities (pink) at **a** side and **b** top views. The solvent accessible volume in the UR-MK299 bound structure (5ZBQ) was calculated with the N-terminal region (18–30) of Y_1R truncated. Each calculated cavity volume is shown below each figure.

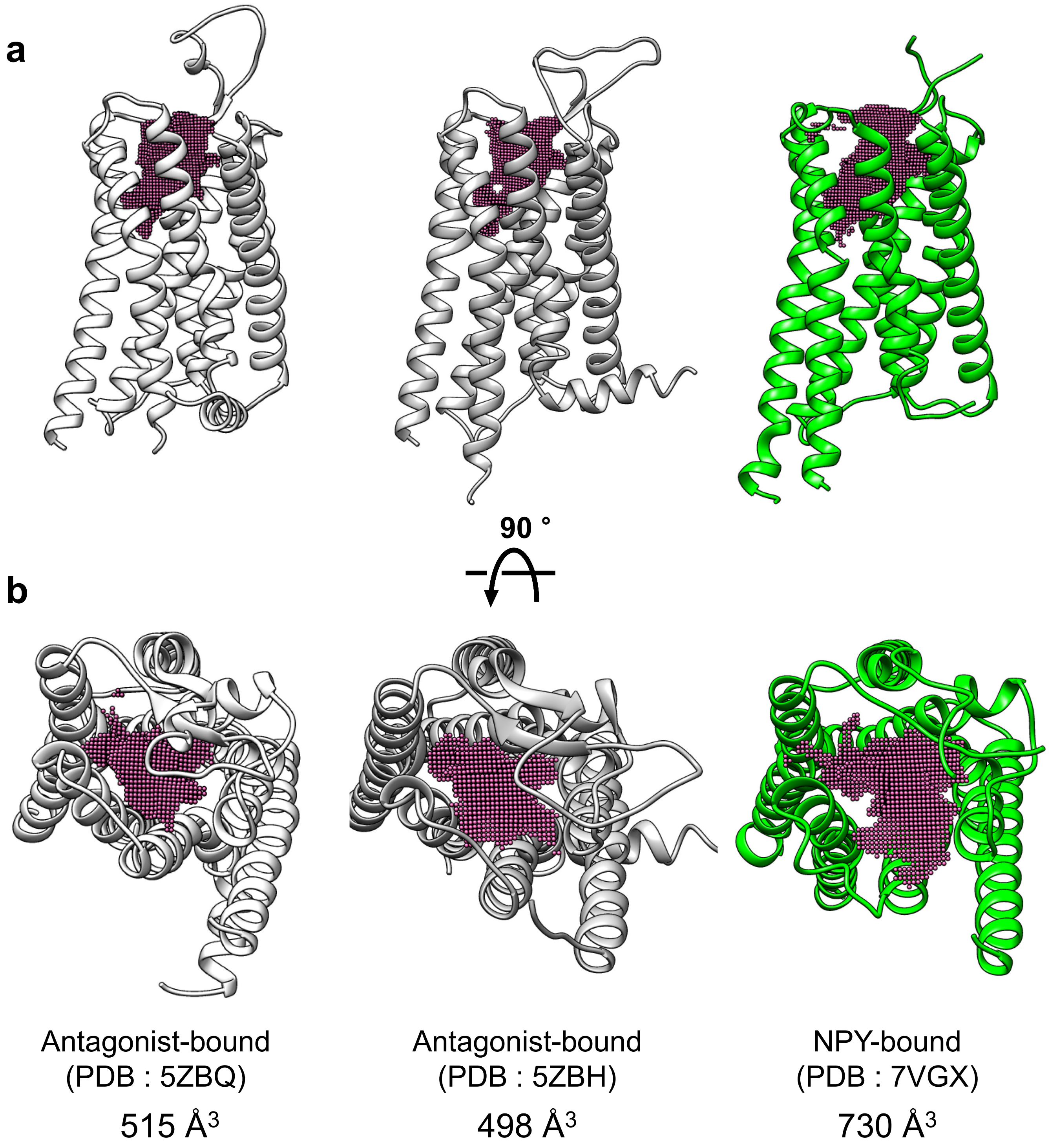

Supplementary Figure 6 Continuous density of the N-terminal region of Y_1R beyond TM1. Density of Y_1R (green), NPY (yellow), cropped G_{i1} (white) and detergent micelles (transparent) from **a** the unsharpened cryo-EM map of the unmasked NPY–Y_1R–G_{i1}–scFv16 complex and **b** local sharpened[3] map focused on the transmembrane domain. Cryo-EM densities corresponding to the N-terminus extending from TM1, ECL2, and ECL3 are indicated by arrows.

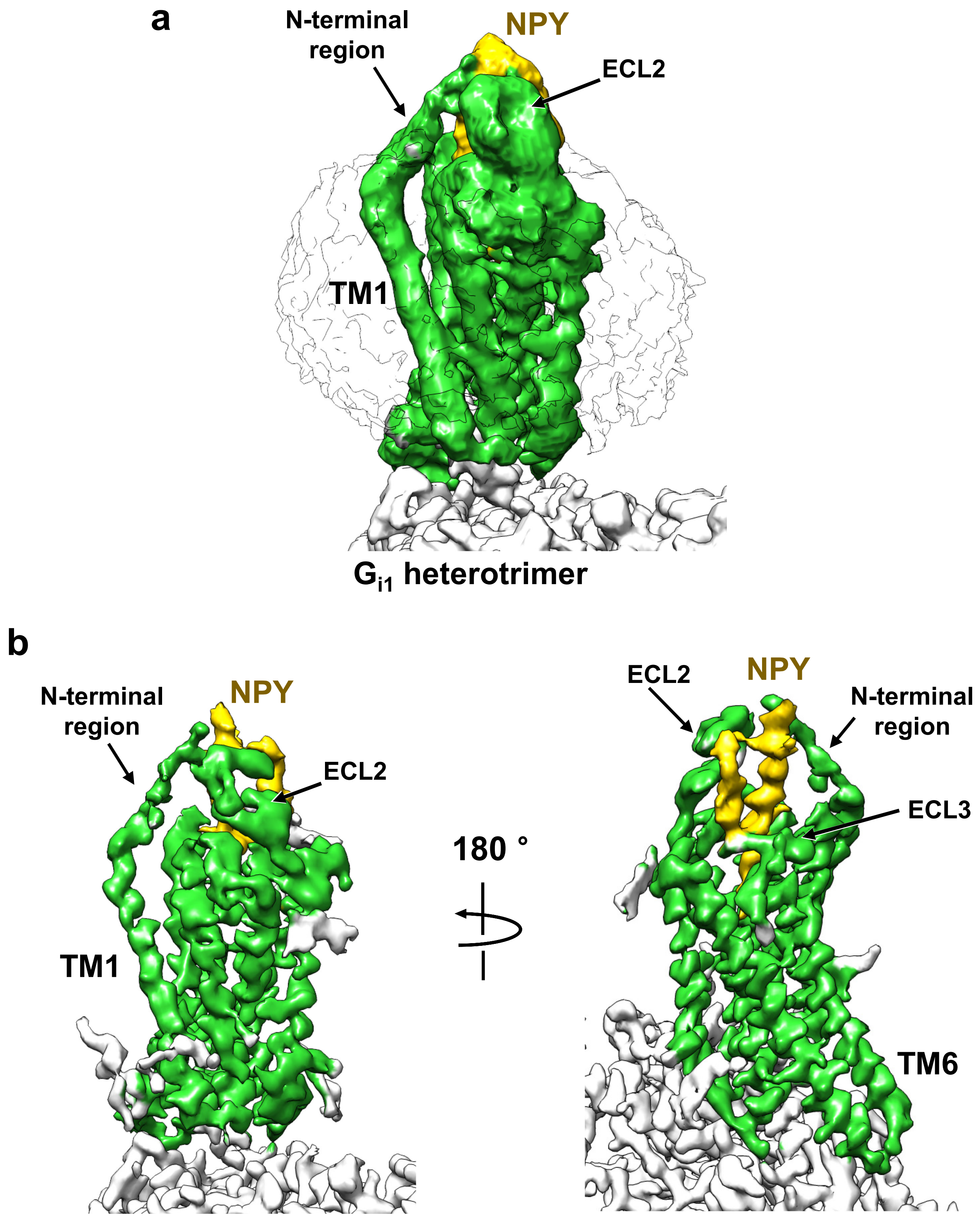

Supplementary Figure 7 G_i binding interface. a G_i binding interfaces were compared among three different GPCR–G_i complexes, Y_1R–G_{i1} (current, PDB ID 7VGX), NTSR1–G_{i1} (PDB ID 6OS9), and μOR–G_{i1} (PDB ID 6DDE) by aligning TM helices. The α5 helix angles of G_i are different from each other by 5–10˚. **b-d** Most interactions between GPCR TMs and G_i are conserved in all three structures, but ICL2-mediated interactions are different.

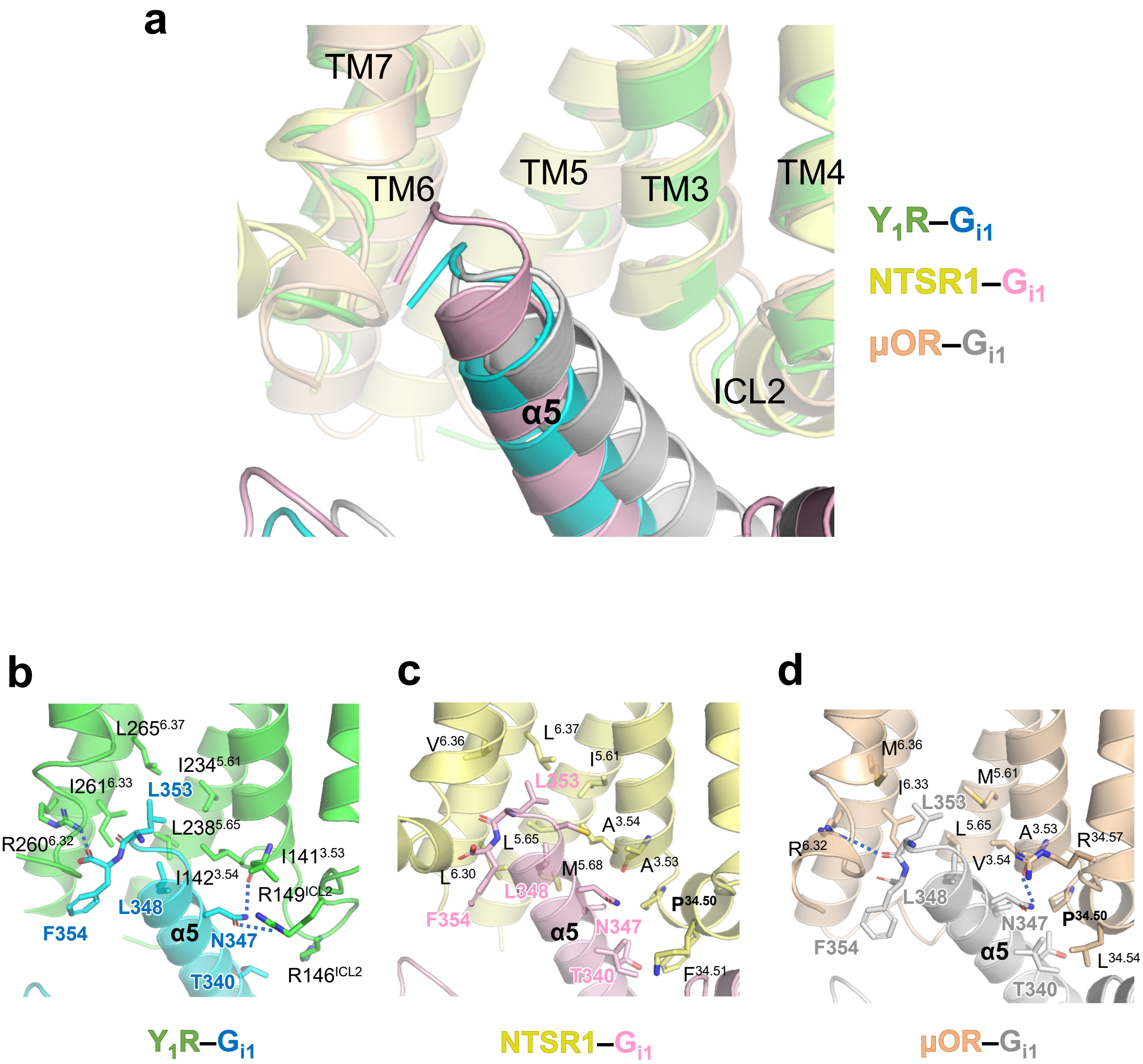

Supplementary Figure 8 BRET and Ca^{2+} signaling assays of NPY and carboxyl-NPY (NPY-COOH). a BRET assays showed that unlike NPY, NPY-COOH does not induce recruitment of G_{i1} to Y_1R. Bar and error bar for each sample represent the mean and S.E.M. of n=six independent experiments, respectively. Each circle represents an individual data point from the experiment. **b** Y_1R does not respond to NPY-COOH at 1 μM concentration, as measured by Ca^{2+} signaling assay. The induction fold by treatment of PBS (black), NPY (blue), or NPY-COOH (red) is shown. The symbol and error bar indicate the mean and S.E.M. of n=three independent experiments for each time point, respectively. Source data are provided as a Source Data file.

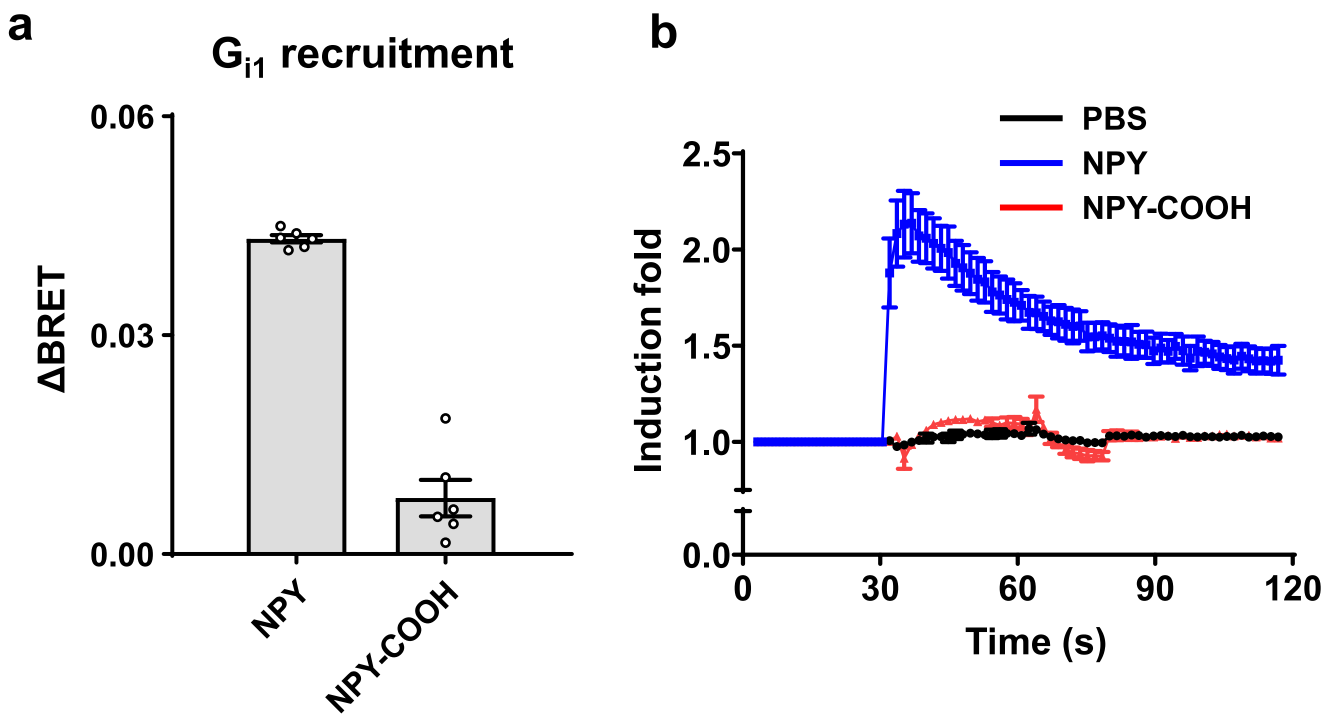

Supplementary Figure 9 Surface expression levels of Y_1R mutants. Each Y_1R mutant displays expression level similar to wild-type. **a** Surface expression levels are analyzed with surface ELISA. Bar and error bar for each sample represent the mean and S.E.M. (standard error of mean) of n=three independent experiments, respectively. Each circle represents an individual data point from the experiment. Source data are provided as a Source Data file. **b** Representative live-cell fluorescence microscopy images of each Y_1R mutant in transiently transfected HEK293 cells are shown (scale bars, 10 μm). Three fields of view were randomly chosen in each confocal dish using DAPI channel to avoid bias in cell selection.

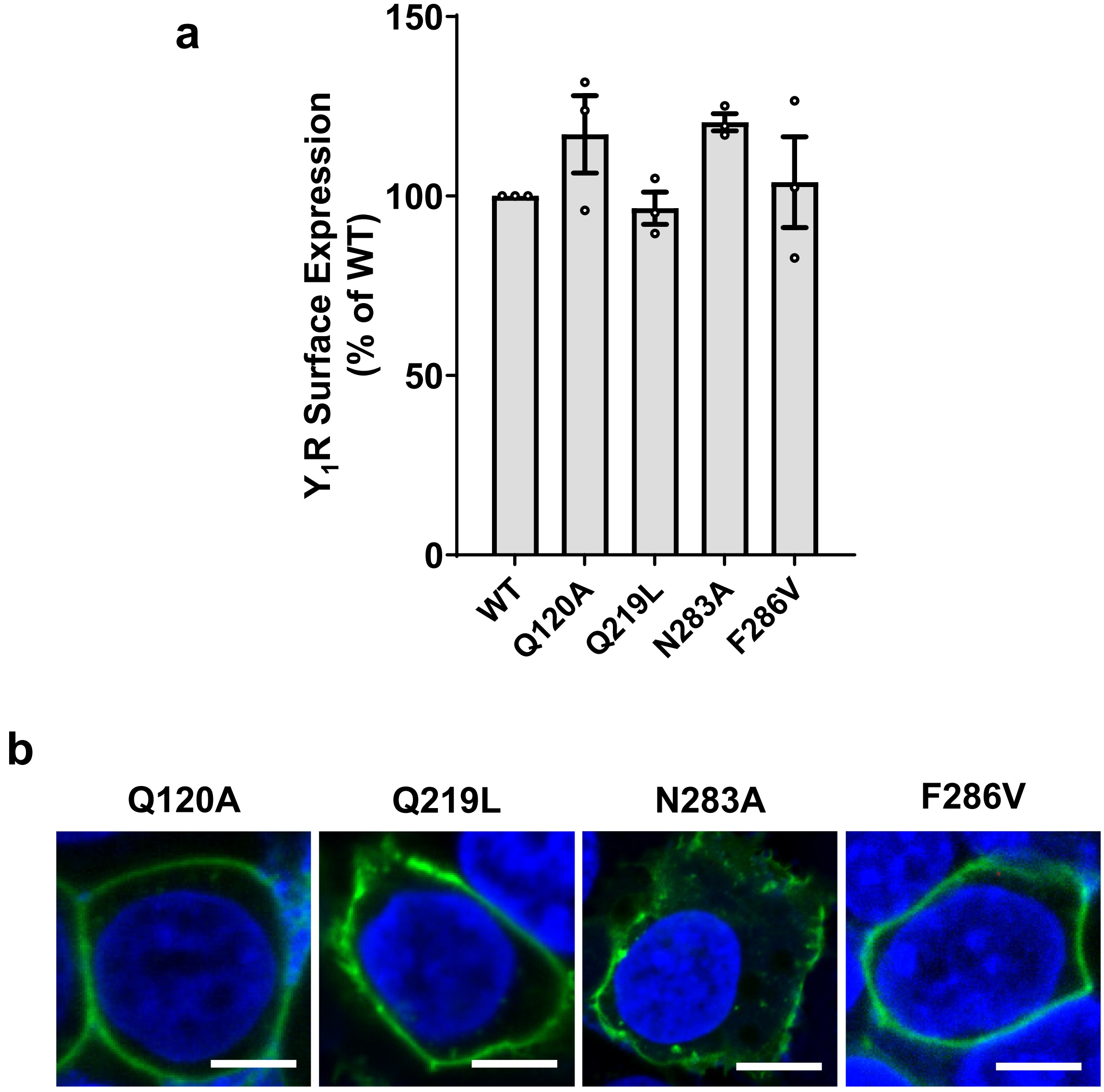

Supplementary Figure 10 G_{i1} recruitment assays of Y_1R mutants. NPY-induced G_{i1} recruitment of Y_1R wild-type (WT) and mutants was measured by BRET assay. 10 nM NPY was treated in each experiment. Bar and error bar for each sample represent the mean and S.E.M. (standard error of mean) of n=six independent experiments, respectively. Each circle represents an individual data point from the experiment. Data analysis was done with one-way ANOVA followed by Tukey's test. '****' means calculated p-value is lower than 0.0001 (between WT and mutants). Exact p-values for other pairs were 0.0146 (between Q120A and Q219L), 0.9974 (between Q120A and N283A), >0.9999 (between Q120A and F286V), 0.0046 (between Q219L and N283A), 0.0149 (between Q219L and F286V),and 0.9972 (between N283A and F286V), respectively. Source data are provided as a Source Data file.

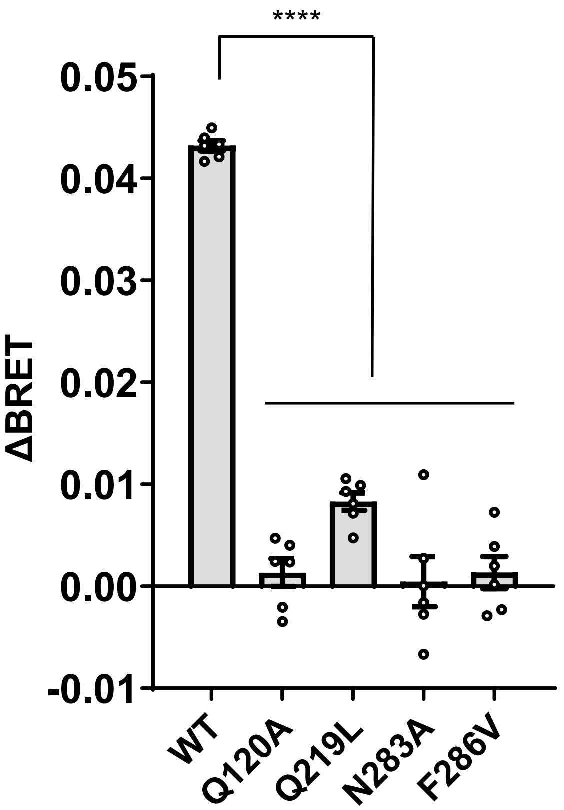

Supplementary Figure 11 Calcium signaling assays of Y_1R mutants. Ca^{2+} assays were performed at 8 different concentrations of NPY for each experiment. Dose response curves were generated by Graphpad Prism 9.2.0 (GraphPad Software, Inc.). Each mutant data (red) is shown along with wild-type data (black) for comparison. Symbol and error bar indicate the mean and S.E.M. of n=three (Q120A, Q219L, N283A), four (F286V) or 17 (WT) independent experiments, respectively. The calculated EC_{50} values (pEC_{50} ± SEM) are provided in **Supplementary Table 2**. Source data are provided as a Source Data file.

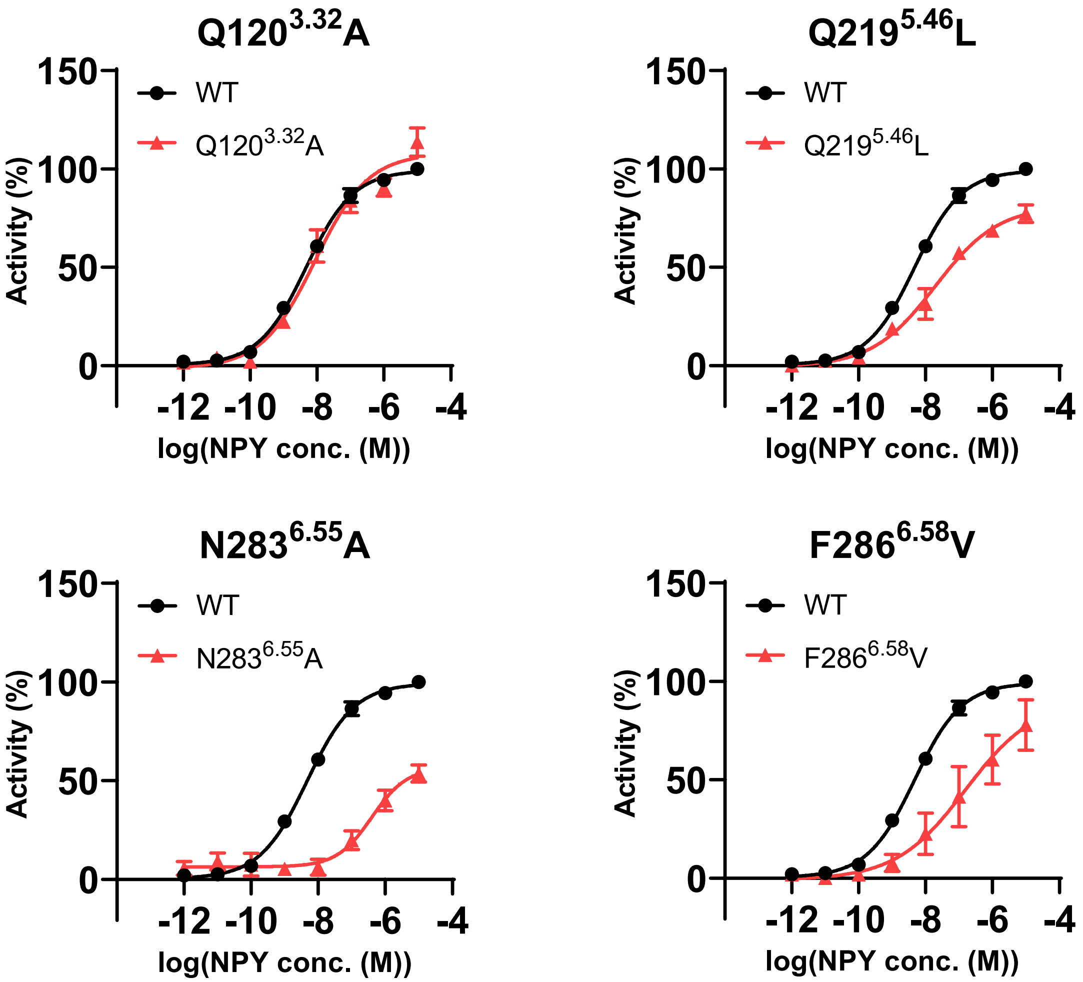

Supplementary Figure 12 Electrostatic surface potentials of GPCRs bound to their peptide ligands. Electrostatic surface potentials of **a** NPY bound Y_1R, **b** Orexin-B bound OX_2R, **c** Endothelin-1 bound ET_BR, and **d** Neurotensin bound NTSR1 were calculated by the CHARM-GUI PBEQ solver and are displayed using PyMOL[4-7]. Red and blue represent negatively and positively charged regions, respectively. Each peptide ligand is represented as sticks and each receptor is represented as cartoon. The amidated C-terminus (NPY, OxB) and carboxyl C-terminus (ET1, NTS) of the peptide ligands are marked with black circles.

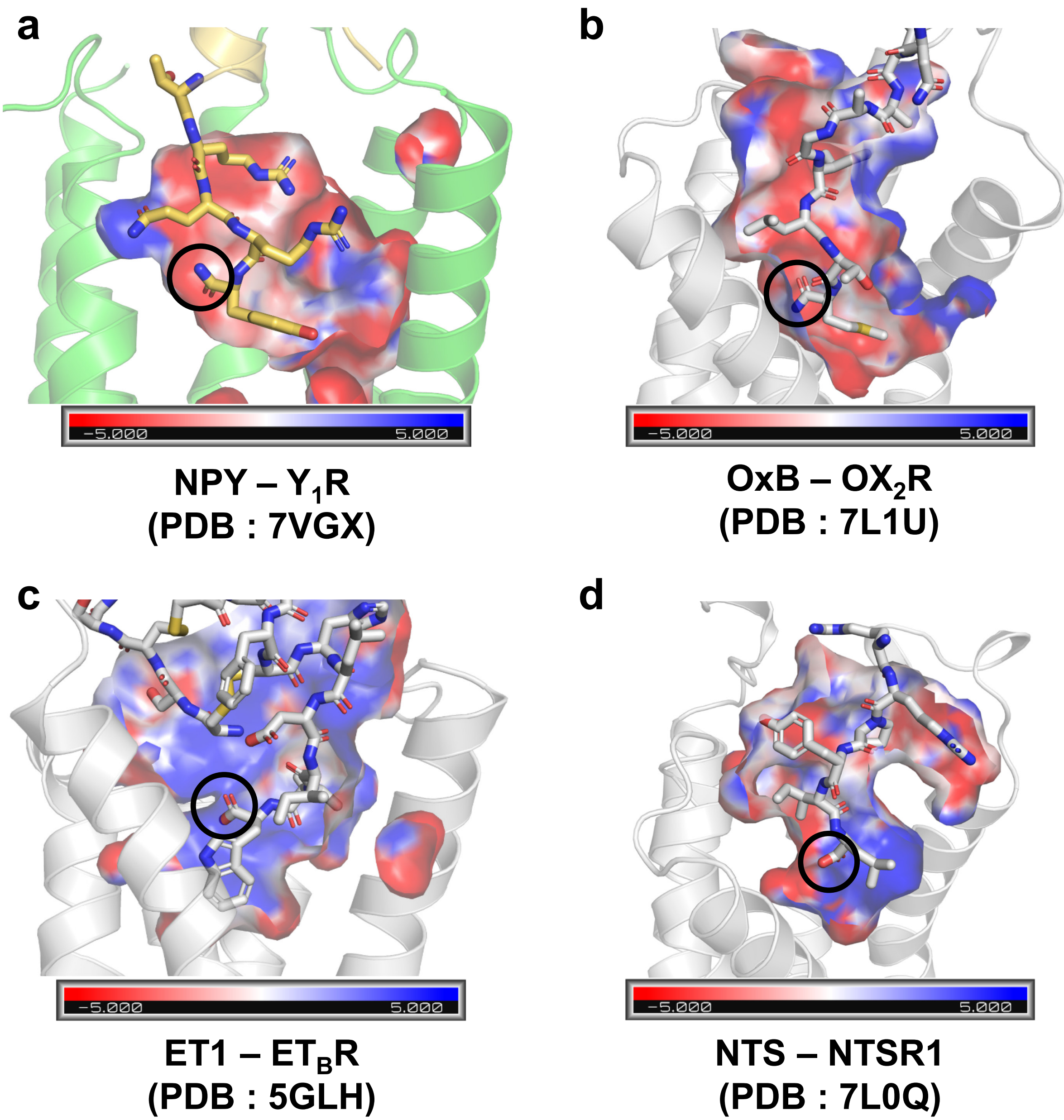

Supplementary Figure 13 Intramolecular interaction between R35 and Y36 of NPY. R35 and Y36 are within van der Waals contact (indicated by dashed lines, < 4 Å) and are represented as stick figures. NPY and Y_1R are colored yellow and green, respectively, as shown in **Figure 1**.

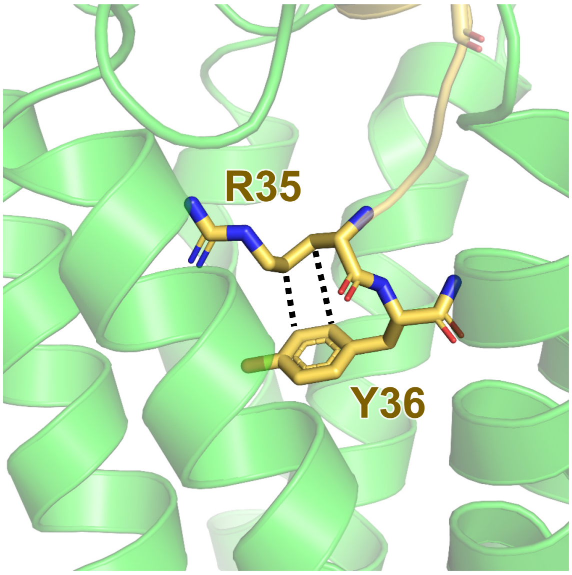

Supplementary Figure 14 Molecular dynamics of the helical region and the N-terminus of NPY. a The tilt angle is defined as the angle between the axis of the NPY α-helix (black line) and the membrane normal axis (red line) as shown on the left. Tilt angles calculated from all replicates are plotted as the simulation time point. The blue dashed line indicates the initial tilt angle (31°). **b** The Cα distances between NPY Y1 and Y_1R D205$^{5.32}$ located at the tip of TM5 were monitored over the course of simulations. The blue dashed line indicates the initial distance (8 Å). Source data for the graphs are provided as a Source Data file.

a

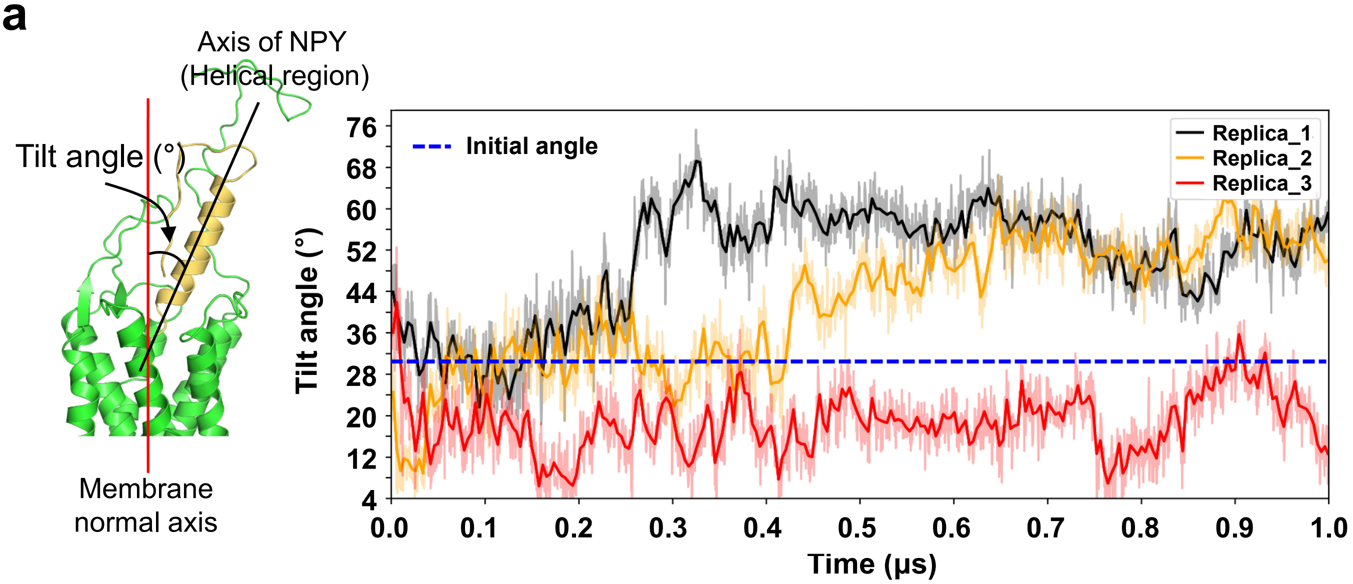

b

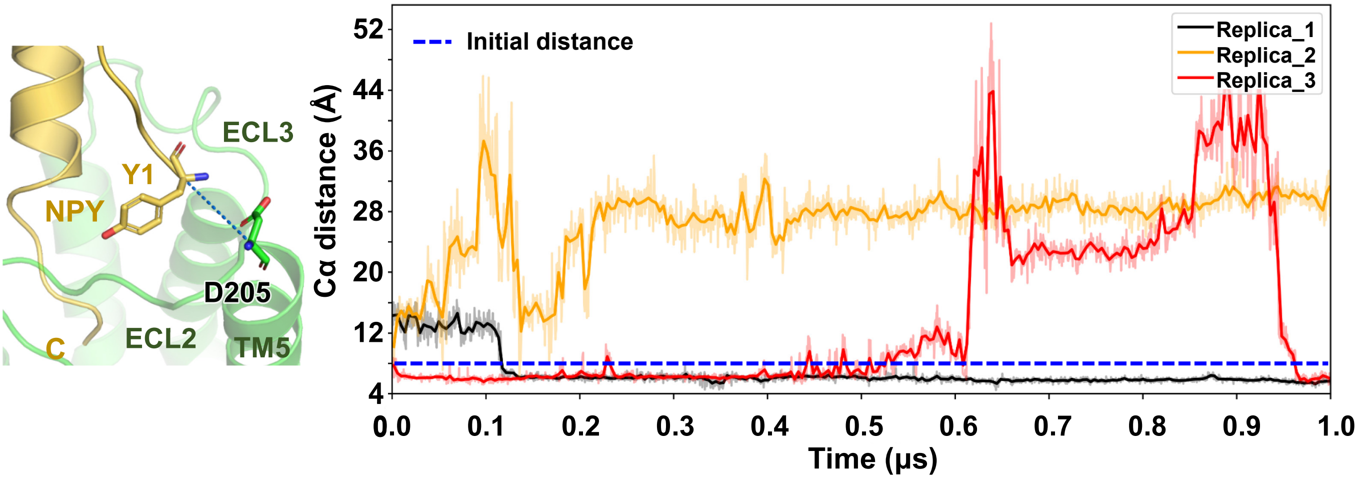

Supplementary Figure 15 Surface expression levels of Y_1R mutants. Each Y_1R mutant displays expression level similar to wild-type. **a** Surface expression levels were analyzed with surface ELISA. Bar and error bar for each sample represent the mean and S.E.M. of n=three independent experiments, respectively. Each circle represents an individual data point from the experiment. Source data are provided as a Source Data file. **b** Representative live-cell fluorescence microscopy images of each Y_1R mutant in transiently transfected HEK293 cells are shown (scale bars, 10 μm). Three fields of view were randomly chosen in each confocal dish using DAPI channel to avoid bias in cell selection.

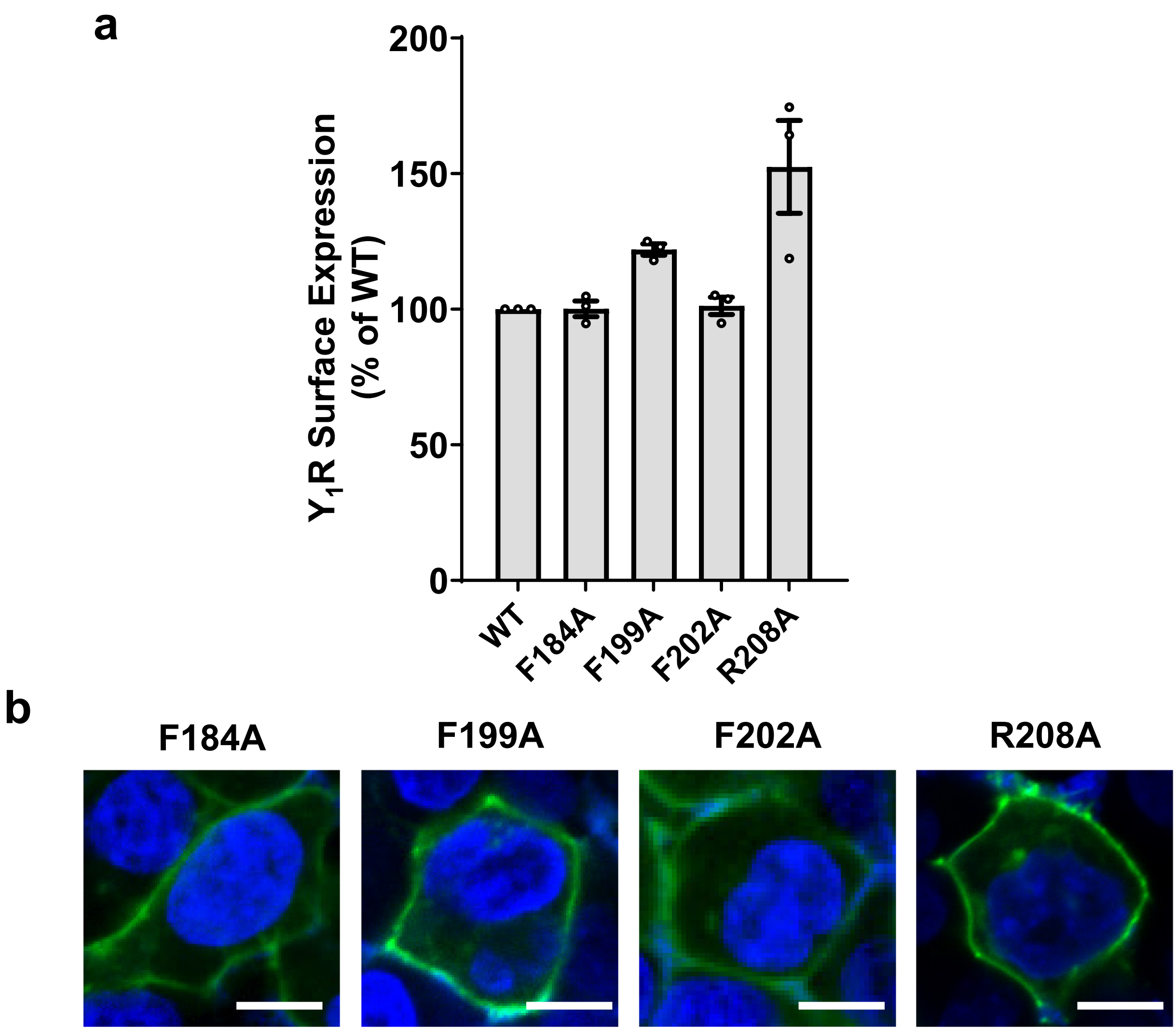

Supplementary Figure 16 G_{i1} recruitment assays of Y_1R mutants. NPY-induced G_{i1} recruitment of Y_1R wild-type (WT) and mutants was measured by BRET assay. 10 nM NPY was treated in each experiment. Bar and error bar for each sample represent the mean and S.E.M. of n=six independent experiments, respectively. Each circle represents an individual data point from the experiment. Data analysis was done with one-way ANOVA followed by Tukey's test. '****' means calculated p-value is lower than 0.0001 (between WT and mutants). Exact p-values for other pairs were <0.0001 (between F202A and other mutants), 0.9673 (between F184A and F199A), 0.9463 (between F184A and R208A), >0.9999 (between F199 and R208A), respectively. Source data are provided as a Source Data file.

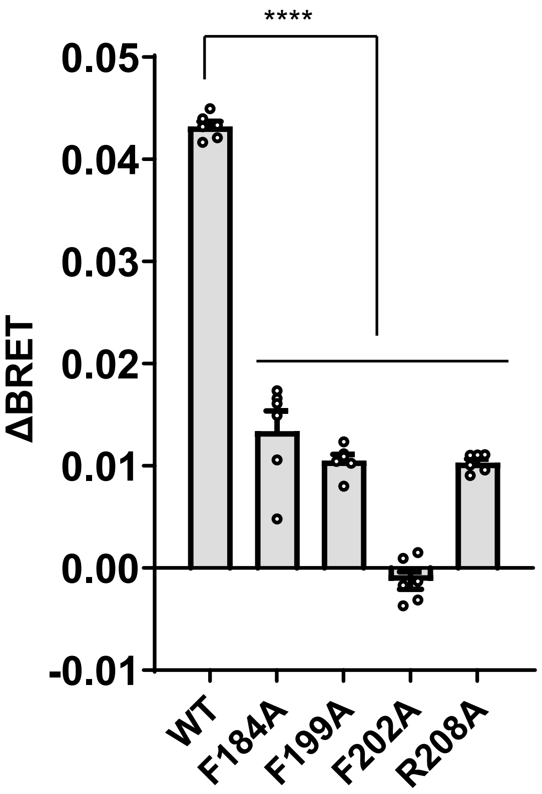

Supplementary Figure 17 Calcium signaling assays of Y_1R mutants. Ca^{2+} assays were performed at 8 different concentrations of NPY for each experiment. Dose response curves were generated by Graphpad Prism 9.2.0 (GraphPad Software, Inc.). Each mutant data (red) is shown along with wild-type data (black) for comparison. Symbol and error bar indicate the mean and S.E.M. of n=three (mutants) or 17 (WT) independent experiments, respectively. The calculated EC_{50} values (pEC_{50} ± SEM) are provided in **Supplementary Table 2**. Source data are provided as a Source Data file.

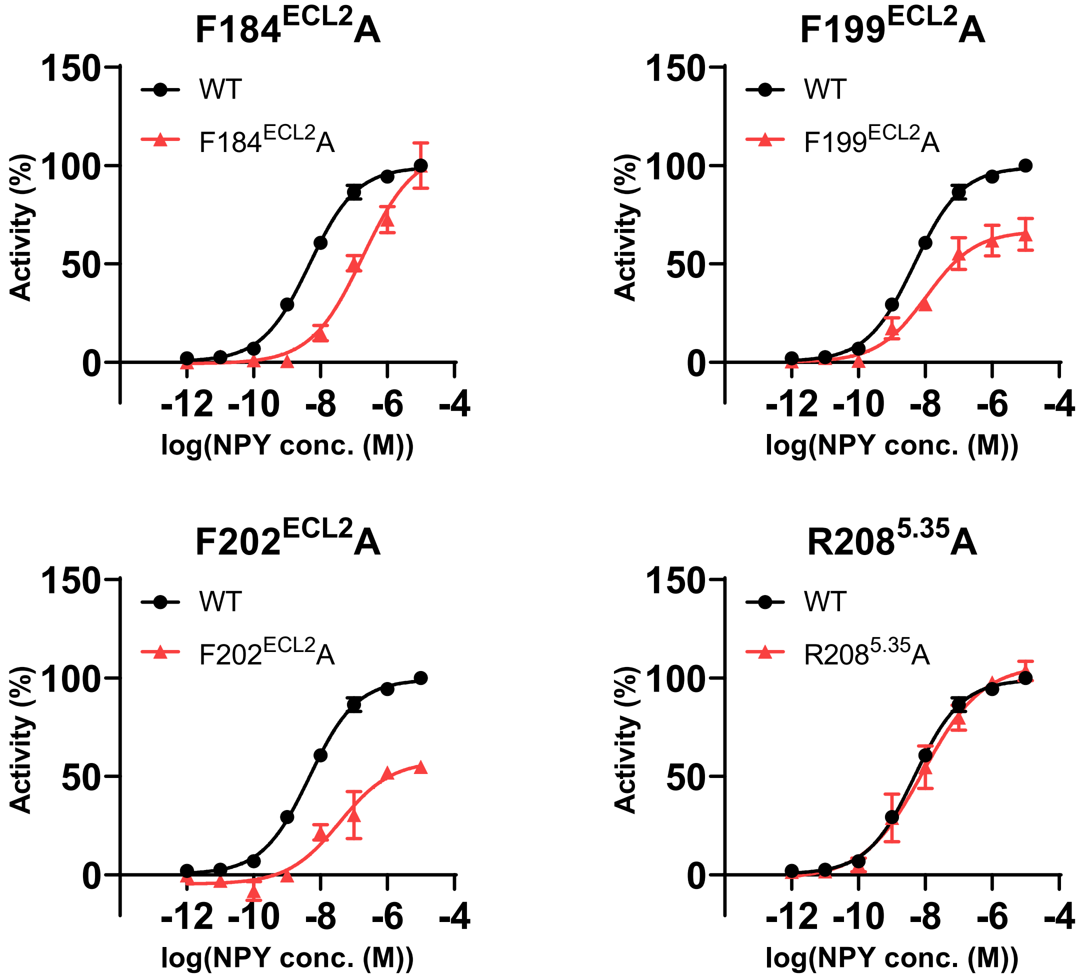

Supplementary Figure 18 F202 contributes to the structural integrity of Y_1R. The aromatic Y_1R residues, $F173^{4.60}$, $Y176^{4.63}$, and $Y211^{5.38}$, make close contact with $F202^{ECL2}$, maintaining structural integrity in both antagonist- and NPY-bound structures. The color code is the same as in **Figure 4.**

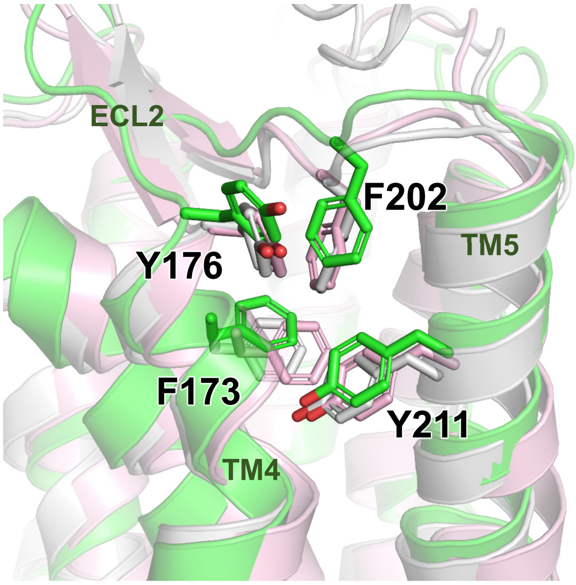

Supplementary Figure 19 Cryo-EM density of NPY peptide. Cryo-EM density of the current NPY model is displayed by UCSF Chimera[8] with a threshold of (**a**) 0.72 and (**b**) 0.52. The continuous density at a lower threshold was used to build the missing NPY residues (6–19) for MD simulations.

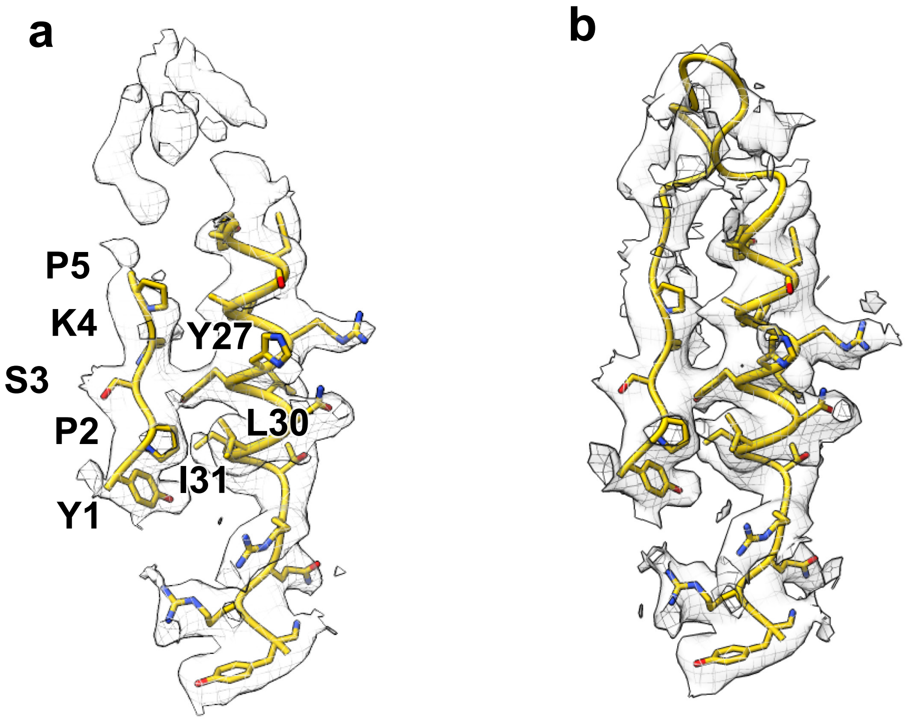

Supplementary Figure 20 Surface expression levels of N-terminal mutants of Y_1R. Each Y_1R mutant displays expression level similar to wild-type. **a** Surface expression levels are analyzed with surface ELISA. Bar and error bar for each sample represent the mean and S.E.M. of n=three independent experiments, respectively. Each circle represents an individual data point from the experiment. Source data are provided as a Source Data file. **b** Representative live-cell fluorescence microscopy images of each Y_1R mutant in transiently transfected HEK293 cells are shown (scale bars, 10 μm). Three fields of view were randomly chosen in each confocal dish using DAPI channel to avoid bias in cell selection.

a

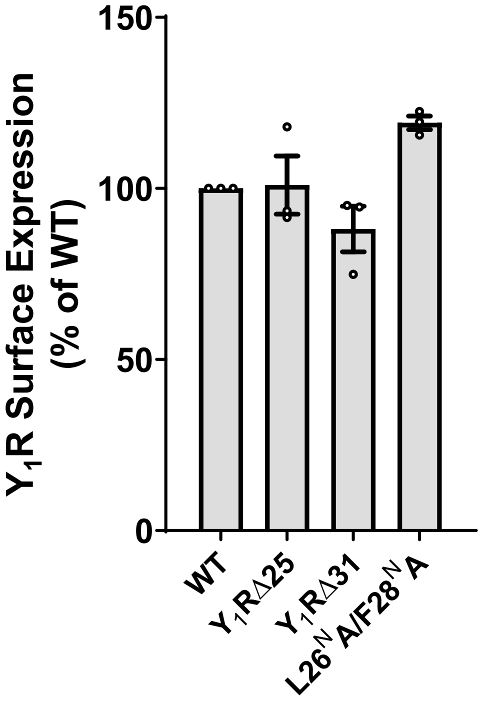

b

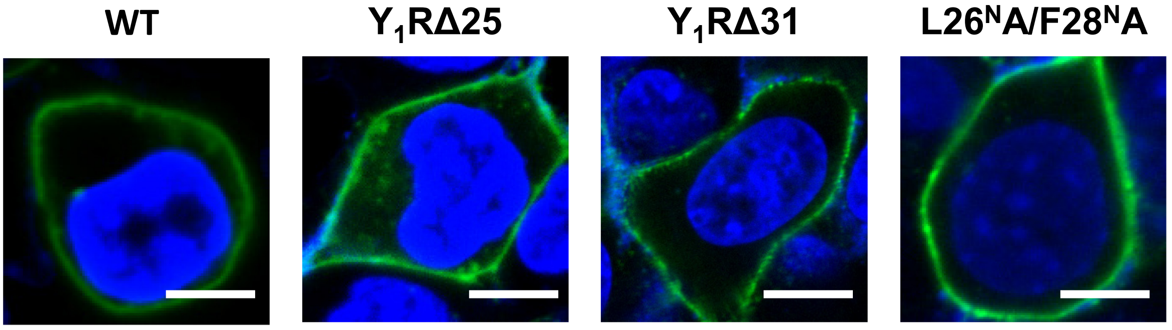

Supplementary Figure 21 G_{i1} recruitment assay of N-terminal mutants of Y_1R. NPY-induced G_{i1} recruitment of Y_1R wild-type (WT) and mutants was measured by BRET assay. 10 nM NPY was treated in each experiment. Bar and error bar for each sample represent the mean and S.E.M. of n=six independent experiments, respectively. Each circle represents an individual data point from the experiment. Data analysis was done with one-way ANOVA followed by Tukey's test. '****' means calculated p-value is lower than 0.0001 (between WT and $Y_1R\Delta31$, WT and $L26^NA/F28^NA$, $Y_1R\Delta25$ and $L26^NA/F28^NA$). Exact p-values for pairs marked as 'NS (Not Significant)' were 0.6891 (between WT and $Y_1R\Delta25$) and 0.3037 (between $Y_1R\Delta31$ and $L26^NA/F28^NA$), respectively. Source data are provided as a Source Data file.

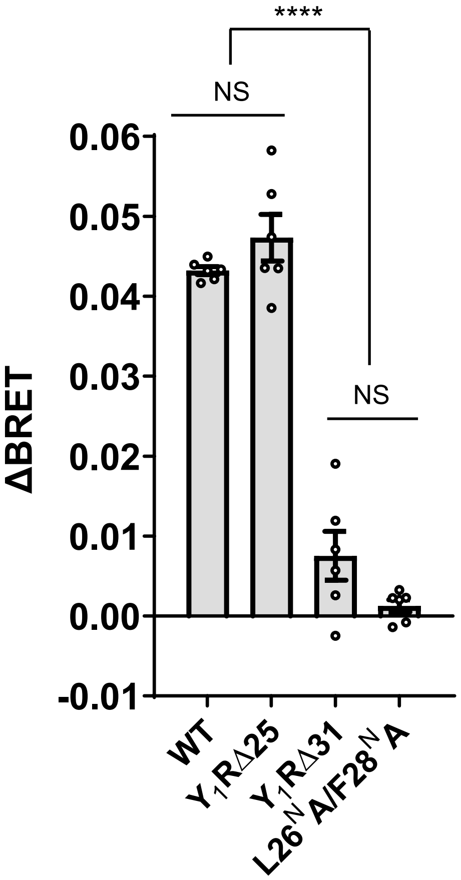

Supplementary Figure 22 Calcium signaling assays of Y_1R mutants. Ca^{2+} assays were performed at 8 different concentrations of NPY for each experiment. Dose response curves were generated by Graphpad Prism 9.2.0 (GraphPad Software, Inc.). Each mutant data (red) is shown along with wild-type data (black) for comparison. Symbol and error bar indicate the mean and S.E.M. of n=three (mutants) or 17 (WT) independent experiments, respectively. The calculated EC_{50} values (pEC_{50} ± SEM) are provided in **Supplementary Table 2**. Source data are provided as a Source Data file.

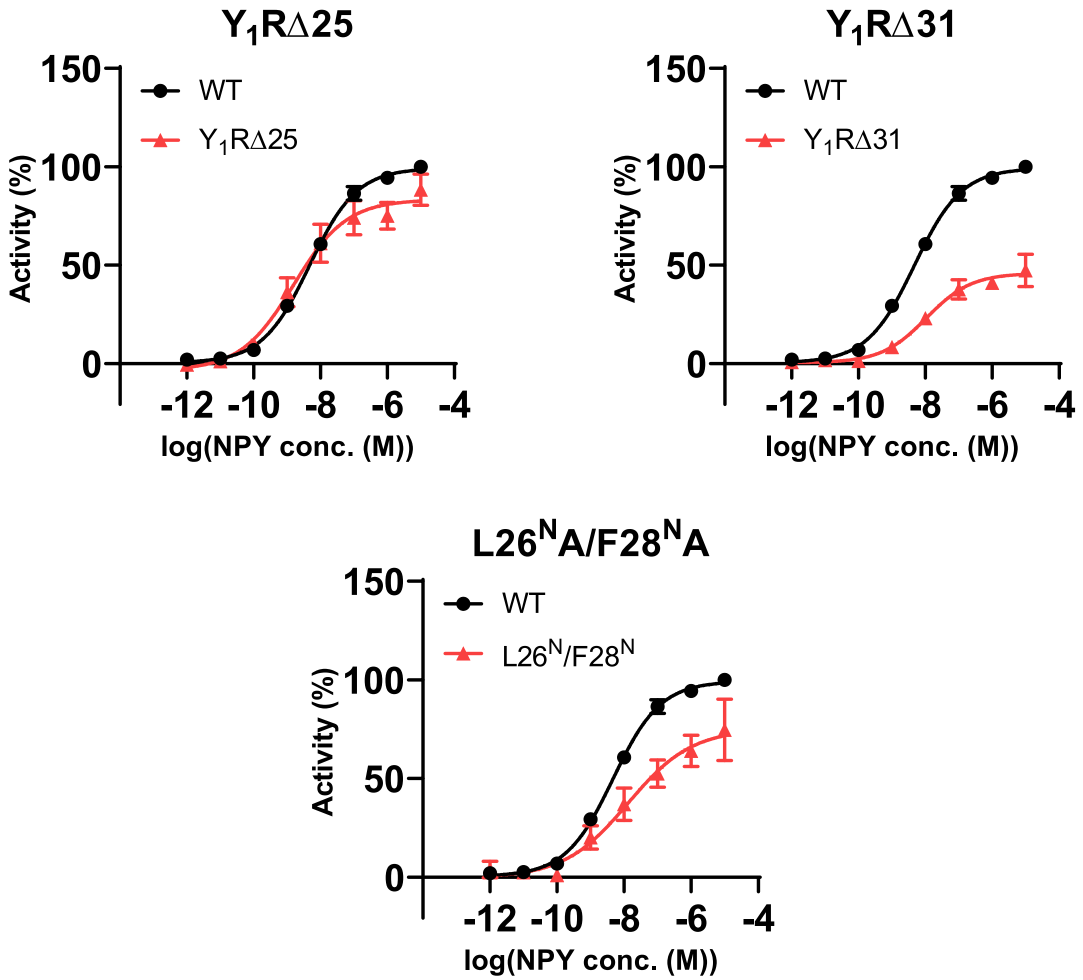

Supplementary Figure 23 Molecular dynamics of the N-terminal region and ECL2 of Y_1R. The Cα distances between Y21 of NPY and **a** $L26^N$ and **b** $F28^N$ during the simulations are plotted. **c** The minimum Cα distance between the helical region of NPY (20–31) and ECL2 of Y_1R (176–205) at each time point is plotted during the simulations. Each blue dashed line indicates the initial distance of each pair (Y21 ~ $L26^N$, 9 Å; Y21 ~ $F28^N$, 13 Å; ECL2 ~ NPY helical region, 3.3 Å). Source data are provided as a Source Data file.

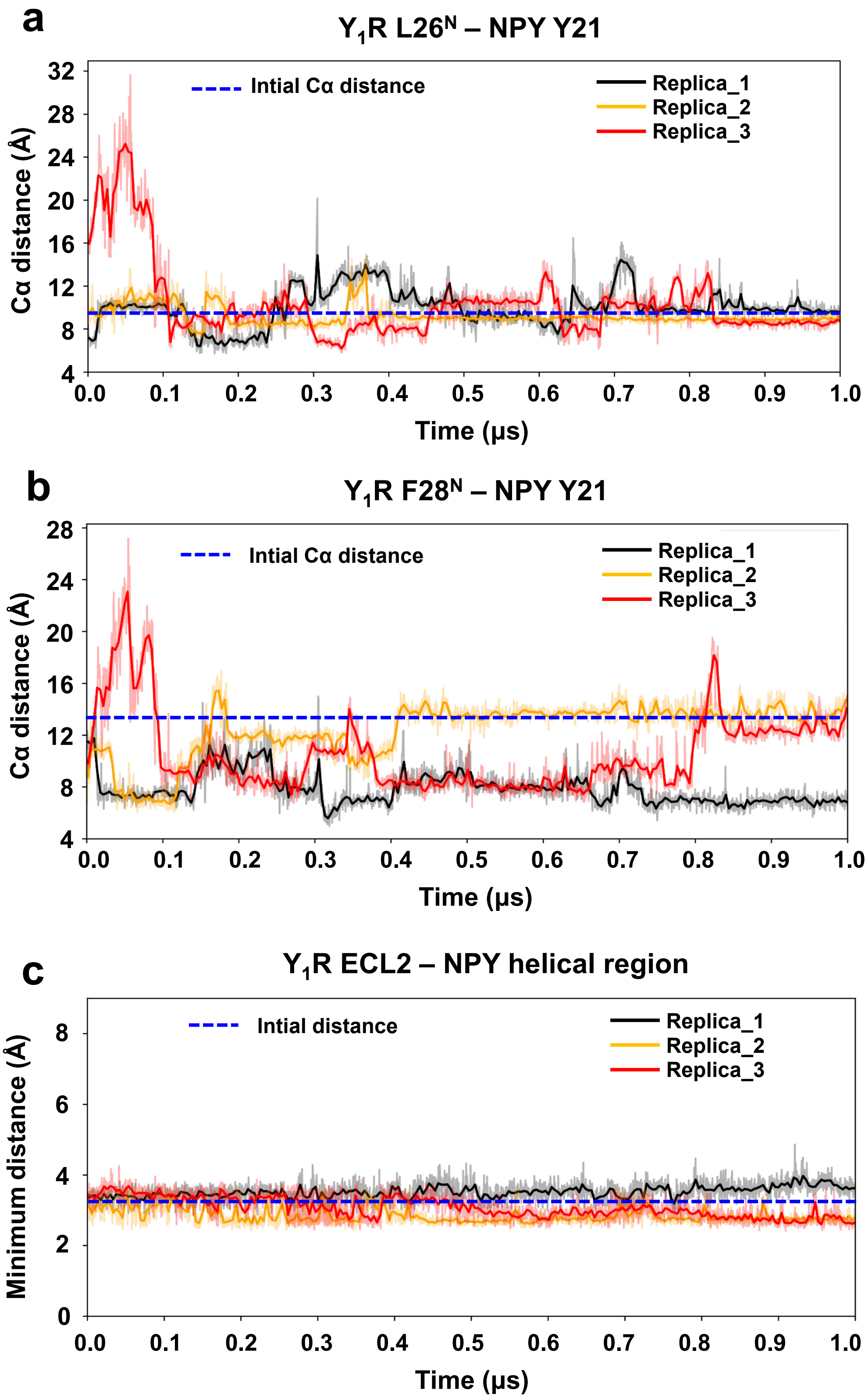

Supplementary Figure 24 Comparison of peptide agonist-bound GPCR structures. The structures of NPY (yellow), NTS (8–13, brown), OxB (teal), CCK-8 (pink) and their receptors (Y_1R in green, others in pale green) are shown. The ligand-binding depth from the top surface of the membrane is similar for NPY, OxB, and CCK-8, but shallower for NTS. Unlike other structures, our current structure shows, at least in part, the extracellular region of NPY.

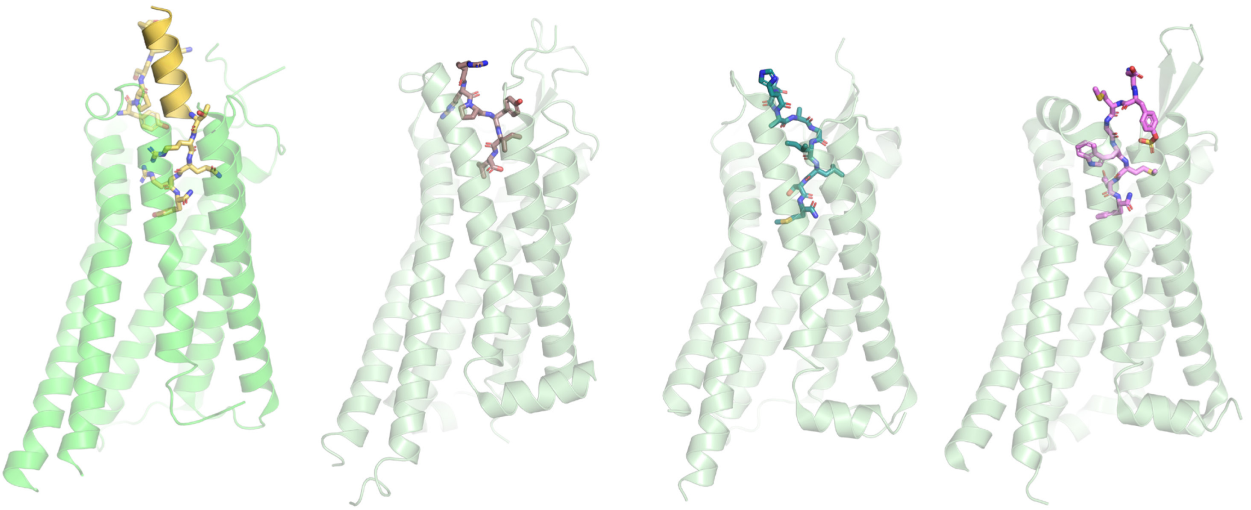

NPY–Y_1R (7VGX)

NTS–NTSR1 (7L0Q)

OxB–OX_2R (7L1U)

CCK-8–CCK1R (7MBX)

Supplementary Figure 25 Sequence alignment of human neuropeptide Y (hNPY), peptide YY (hPYY), and pancreatic polypeptide (hPP). NPY has 67% and 50% sequence identity to PYY and PP, respectively. Conserved residues among three peptides are highlighted in blue.

```
     1         11        21        31
hNPY YPSKPDNPGEDAPAEDMARYYSALRHYINLITRQRY-NH2
hPYY YPIKPEAPREDASPEELNRYYASLRHYLNLVTRQRY-NH2
hPP  APLEPVYPGDNATPEQMAQYAADLRRYINMLTRPRY-NH2
```

Supplementary Figure 26 Calcium signaling assays with PYY, PYY(3–36), and PP. EC_{50} values of **a** full-length PYY, truncated PYY(3–36), and **b** PP were measured by Ca^{2+} signaling assays. Each dose response curve was fitted by Graphpad Prism 9.2.0 (GraphPad Software, Inc.) and the calculated EC_{50} values are represented. Symbol and error bar indicate the mean and S.E.M. of n= four (PP), five (PYY, PYY(3-36)) or 17 (NPY) independent experiments, respectively. The calculated EC_{50} values (pEC_{50} ± SEM) are provided in **Supplementary Table 2.** Source data are provided as a Source Data file.

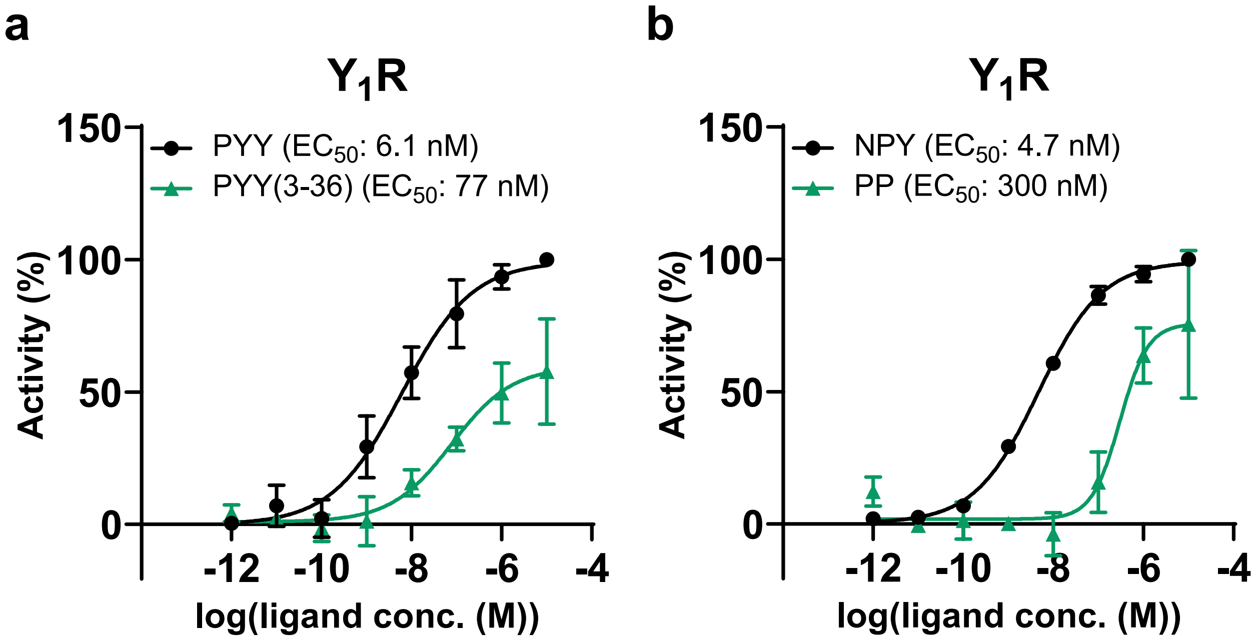

Supplementary Figure 27 Structure-based sequence alignment of four subtypes of NPY receptor. The sequence alignment between human Y_1R, Y_2R, Y_4R, and Y_5R is shown (aligned by alignment tool provided by GPCRdb[9, 10]). The TM helices in the current Y_1R structure are indicated by the green coils. Residues that interact with the C-terminal tail of NPY are highlighted in yellow, residues that interact with the N-terminal loop and helical region of NPY are highlighted in blue, and residues that interact with other regions of NPY together with the C-terminal tail are highlighted in orange.

```
                                                                   TM1
NPY1R ------------MNSTLFSQVENHSVHSNFSEKNAQLLAFENDDCHLPLAMIFTLALAYGAVIILGVSGNLALIIIIL   66
NPY2R MGPIGAEADENQTVEEMKVEQYGPQTTPRGELVPDPEPELIDSTKLI--EVQVVLILAYCSIILLGVIGNSLVIHVVI   76
NPY4R -----------MNTSHLLALLLPKSPQGENRSKPLGTPYNFSEHCQDSVDVMVFIVTSYSIETVVGVLGNLCLMCVTV   67
NPY5R ---------MDLELDEYYNKTLATENNTAATRNSDFPVWDDYKSSVD--DLQYFLIGLYTFVSLLGFMGNLLILMALM   67

                     TM2                                           TM3
NPY1R KQKEMRNVTNILIVNLSFSDLLVAIMCLPFTFVYTLMDHWVFGEAMCKLNPFVQCVSITVSIFSLVLIAVERHQLIIN  144
NPY2R KFKSMRTVTNFFIANLAVADLLVNTLCLPFTLTYTLMGEWKMGPVLCHLVPYAQGLAVQVSTITLTVIALDRHRCIVY  154
NPY4R RQKEKANVTNLLIANLAFSDFLMCLLCQPLTAVYTIMDYWIFGETLCKMSAFIQCMSVTVSILSLVLVALERHQLIIN  145
NPY5R KKRNQKTTVNFLIGNLAFSDILVVLFCSPFTLTSVLLDQWMFGKVMCHIMPFLQCVSVLVSTLILISIAIVRYHMIKH  145

                   TM4
NPY1R PRGWRPNNRHAYVGIAVIWVLAVASSLPFLIYQVMTDEPFQNVTLDAYKDKYV--CFD----QFPSDSHRLSYTTLLL  216
NPY2R HLESKISKRISFLIIGLAWGISALLASPLAIFREYSLIEIIPDFEIVA-------CTEKWPGEEK-SIYGTVYSLSSL  224
NPY4R PTGWKPSISQAYLGIVLIWVIACVLSLPFLANSILENVFHKNHSKALEFLADKVVCTE----SWPLAHHRTIYTTFLL  219
NPY5R PISNNLTANHGYFLIATVWTLGFAICSPLPVFHSLVELQETFGSALLSSRYL---CVE---SWPS-DSYRIAFTISLL  216

         TM5
NPY1R VLQYFGPLCFIFICYFKIYIRLKRRNNMMD---KM-------------------------------------------  248
NPY2R LILYVLPLGIISFSYTRIWSKLKNHVSPG----A--------------------------------------------  254
NPY4R LFQYCLPLGFILVCYARIYRRLQRQGRVF----HKG------------------------------------------  251
NPY5R LVQYILPLVCLTVSHTSVCRSISCGLSNKENRLEENEMINLTLHPSKKSGPQVKLSGSHKWSYSFIKKHRRRYSKKTA  294

NPY1R -------------------------------------------------------------RDNKYRSSETKRINIML  265
NPY2R -------------------------------------------------------------A-NDHYHQRRQKTTKML  270
NPY4R ------------------------------------------------------------TY--SLRAGHMKQVNVVL  267
NPY5R CVLPAPERPSQENHSRILPENFGSVRSQLSSSSKFIPGVPTCFEIKPEENSDVHELRVKRSV--TRIKKRSRSVFYRL  370

        TM6                                       TM7
NPY1R LSIVVAFAVCWLPLTIFNTVFDWNHQIIA-TCNHNLLFLLCHLTAMISTCVNPIFYGFLNKNFQRDLQFFF-----  335
NPY2R VCVVVVFAVSWLPLHAFQLAVDIDSQ-VLDLKEYKLIFTVFHIIAMCSTFANPLLYGWMNSNYRKAFLSAFRCEQR  345
NPY4R VVMVVAFAVLWLPLHVFNSLEDWHHEAIP-ICHGNLIFLVCHLLAMASTCVNPFIYGFLNTNFKKEIKALV-----  337
NPY5R TILILVFAVSWMPLHLFHVVTDFNDN-LISNRHFKLVYCICHLLGMMSCCLNPILYGFLNNGIKADLVSLIHCLHM  445

NPY1R NFCDFRSRDDDYETIAMSTMHTDVSKTSLKQASPVAFKKINNNDDNEKI  384
NPY2R LDAIHSEVSVTFKAKKNLEVRKNSGPNDSFTEATNV-------------  381
NPY4R LTCQQSAPLEESEHLPLSTVHTEVSKGSLRLSGRSNPI-----------  375
NPY5R -------------------------------------------------
```

Supplementary Figure 28 Homology modeling of Y_4R in complex with PP. Calculated model structures of Y_4R and PP are colored light brown and green cyan, respectively. The Y_4R residues that are not conserved between Y_1R and Y_4R ($E288^{6.58}$ and $E203^{ECL2}$) are marked in yellow. The Y_4R residues including $E288^{6.58}$ form an extensive polar interaction network with R33, and R35 of PP. Polar interactions are indicated by dashed lines.

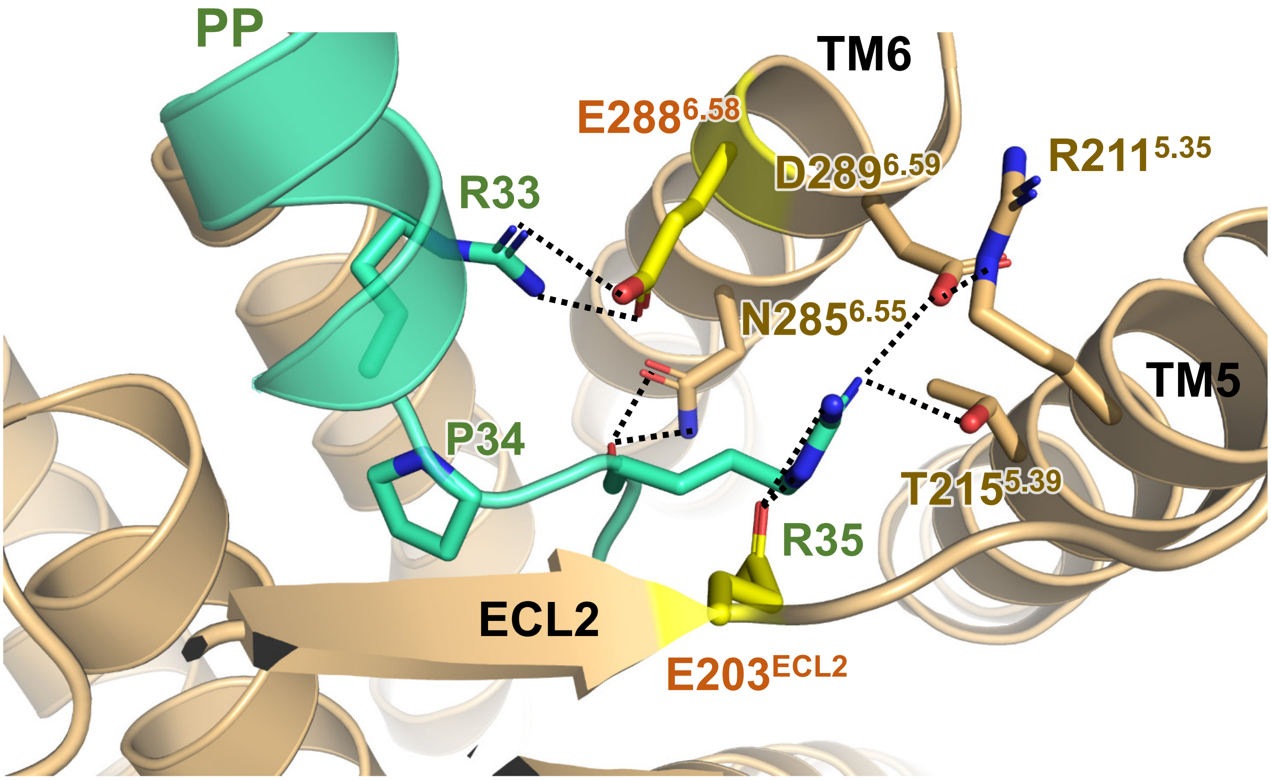

Supplementary Figure 29 GTP turnover assays. GTP turnover assay results are analyzed by Graphpad Prism 9.2.0 (GraphPad Software, Inc.), and the means are displayed as bar graphs. Purified G_{i1} heterotrimers containing $G\alpha_{i1}$ produced from *E.coli* (not lipidated) and Sf9 insect cells, labeled as G_{i1} (*E.coli*) and G_{i1} (Sf9), respectively, were used for GTP turnover assays. G_{i1} (*E.coli*) and G_{i1} (Sf9) exhibit similar GTP hydrolytic activity (95.5% and 95.1% reduced luminescence signals). Three independent experiments were performed, and each data point is represented as a triangle. Source data are provided as a Source Data file.

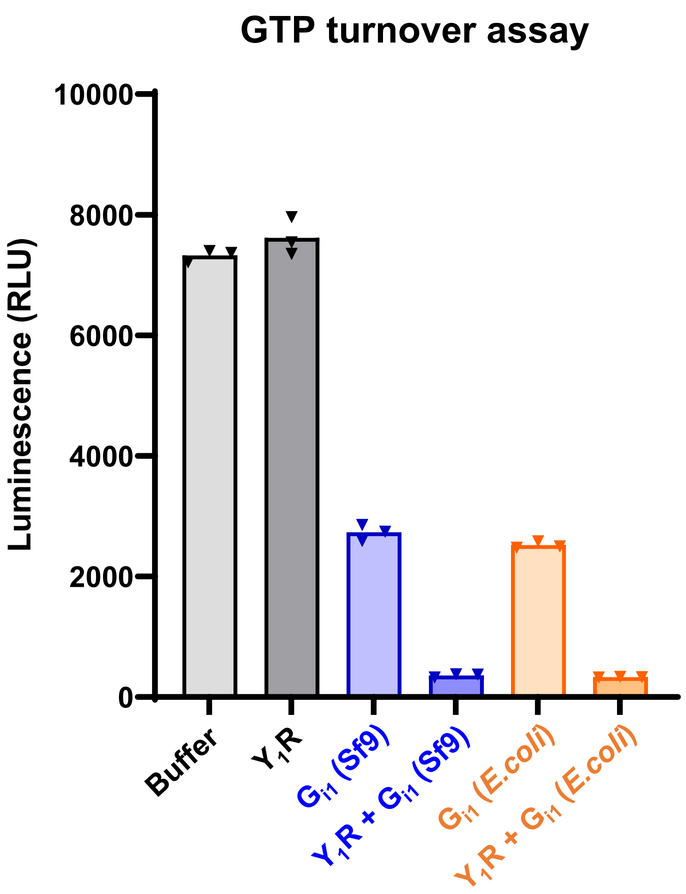

References

1. Williams CJ*, et al.* MolProbity: More and better reference data for improved all-atom structure validation. *Protein Sci.* **27**, 293-315 (2018).

2. Barad BA*, et al.* EMRinger: side chain-directed model and map validation for 3D cryo-electron microscopy. *Nat. Methods* **12**, 943-946 (2015).

3. Kaur S*, et al.* Local computational methods to improve the interpretability and analysis of cryo-EM maps. *Nat. Commun.* **12**, 1240 (2021).

4. Jo S, Kim T, Iyer VG, Im W. CHARMM-GUI: a web-based graphical user interface for CHARMM. *J. Comput. Chem.* **29**, 1859-1865 (2008).

5. Jo S, Vargyas M, Vasko-Szedlar J, Roux B, Im W. PBEQ-Solver for online visualization of electrostatic potential of biomolecules. *Nucleic Acids Res.* **36**, W270-275 (2008).

6. Im W, Beglov D, Roux B. Continuum Solvation Model: computation of electrostatic forces from numerical solutions to the Poisson-Boltzmann equation. *Computer Physics Communications* **111**, 59-75 (1998).

7. Schrödinger. The PyMOL Molecular Graphics System, Version 2.4.0, Schrödinger LLC.) (2020).

8. Pettersen EF*, et al.* UCSF Chimera--a visualization system for exploratory research and analysis. *J. Comput. Chem.* **25**, 1605-1612 (2004).

9. Isberg V*, et al.* GPCRdb: an information system for G protein-coupled receptors. *Nucleic Acids Res* **45**, 2936 (2017).

10. Kooistra AJ*, et al.* GPCRdb in 2021: integrating GPCR sequence, structure and function. *Nucleic Acids Res* **49**, D335-d343 (2021).

Uncropped gel image for Supplementary Figure 2b Uncropped gel image showing the eluted fractions of the NPY–Y_1R–G_i–scFv16 complex from size exclusion chromatography. Each protein band is labeled on the right, and 'inj' indicates the 'injection sample' of the size exclusion chromatography. The cropped image shown in **Supplementary Figure 2b** is indicated by rectangles.

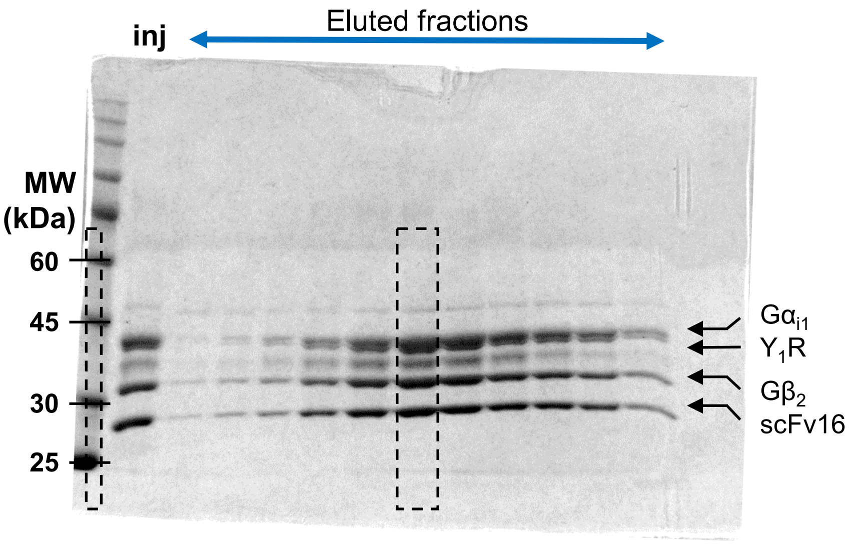

Uncropped gel image
