# Supplementary material for: Structural basis of neuropeptide Y signaling through Y1 receptor
Source: Nat Commun. 2022 Feb 14;13:853. doi: 10.1038/s41467-022-28510-6 (PMC8844075; doi:10.1038/s41467-022-28510-6)
Supplement: Supplementary file 3 — Description of Additional Supplementary Files [file 41467_2022_28510_MOESM3_ESM.pdf]

Description of Additional Supplementary Files

File name: Supplementary Data 1

Description: The python code for calculating the solvent accessible volume of the ligand-binding pocket.
