# Supplementary material for: Structural basis of neuropeptide Y signaling through Y1 receptor
Source: Nat Commun. 2022 Feb 14;13:853. doi: 10.1038/s41467-022-28510-6 (PMC8844075; doi:10.1038/s41467-022-28510-6)
Supplement: Supplementary file 5 — Reporting Summary [file 41467_2022_28510_MOESM5_ESM.pdf]

nature portfolio

Corresponding author(s): Hee-Jung Choi

Last updated by author(s): Jan 7, 2022

Reporting Summary

Nature Portfolio wishes to improve the reproducibility of the work that we publish. This form provides structure for consistency and transparency in reporting. For further information on Nature Portfolio policies, see our Editorial Policies and the Editorial Policy Checklist.

Statistics

For all statistical analyses, confirm that the following items are present in the figure legend, table legend, main text, or Methods section.

n/a	Confirmed	
☐	☒	The exact sample size (*n*) for each experimental group/condition, given as a discrete number and unit of measurement
☐	☒	A statement on whether measurements were taken from distinct samples or whether the same sample was measured repeatedly
☐	☒	The statistical test(s) used AND whether they are one- or two-sided<br>*Only common tests should be described solely by name; describe more complex techniques in the Methods section.*
☒	☐	A description of all covariates tested
☐	☒	A description of any assumptions or corrections, such as tests of normality and adjustment for multiple comparisons
☐	☒	A full description of the statistical parameters including central tendency (e.g. means) or other basic estimates (e.g. regression coefficient) AND variation (e.g. standard deviation) or associated estimates of uncertainty (e.g. confidence intervals)
☐	☒	For null hypothesis testing, the test statistic (e.g. *F*, *t*, *r*) with confidence intervals, effect sizes, degrees of freedom and *P* value noted<br>*Give P values as exact values whenever suitable.*
☒	☐	For Bayesian analysis, information on the choice of priors and Markov chain Monte Carlo settings
☒	☐	For hierarchical and complex designs, identification of the appropriate level for tests and full reporting of outcomes
☒	☐	Estimates of effect sizes (e.g. Cohen's *d*, Pearson's *r*), indicating how they were calculated

Our web collection on statistics for biologists contains articles on many of the points above.

Software and code

Policy information about availability of computer code

Data collection	Cryo-EM data were collected at the Korea Basic Science Institute (KBSI, KOREA), with the EPU package (1.50) installed on the Titan Krios microscope. Data collection and refinement statistics are shown in Supplementary Table 1.
Data analysis	The following softwares were used in this study. cryoSPARC 3.1 (motion-correction, CTF estimation, 2D-averageing, and 3D-refinement), Topaz 0.2.4 (particle picking), CryoSPARC 3.2 (local refinement), Coot 0.9.4, Phenix 1.19, MolProbity 4.5, LocSpiral, Chimera 1.15, ChimeraX 1.2.5, Pymol 2.4.0, GraphPadPrism 9.2.0, ZEN 3.4, GROMACS 2018.6, CHARMM-GUI 3.6, LINCS, PBEQ solver, GalaxyTBM, GalaxyRefineComplex, Galaxy7TM

For manuscripts utilizing custom algorithms or software that are central to the research but not yet described in published literature, software must be made available to editors and reviewers. We strongly encourage code deposition in a community repository (e.g. GitHub). See the Nature Portfolio guidelines for submitting code & software for further information.

Data

Policy information about availability of data

All manuscripts must include a data availability statement. This statement should provide the following information, where applicable:

- Accession codes, unique identifiers, or web links for publicly available datasets
- A description of any restrictions on data availability
- For clinical datasets or third party data, please ensure that the statement adheres to our policy

The cryo-EM density maps have been deposited in the Electron Microscopy Data Bank under accession number of EMD-31979 and the atomic model has been deposited in the Protein Data Bank under accession number of 7VGX. The structures of inactive Y1R (PDB ID 5ZBH), Gαiβγ and scFv16 from the NTSR1 complex

structure (PDB ID 6OS9) were used as an initial template to build the NPY-Y1R-G protein model. Structural models used in data analysis were accessed from the Protein Data Bank under accession numbers of 5ZBQ, 6DDE, 7L1U, 5GLH, 7L0Q and 7MBX. Source data are provided with this paper.

Field-specific reporting

Please select the one below that is the best fit for your research. If you are not sure, read the appropriate sections before making your selection.

☒ Life sciences ☐ Behavioural & social sciences ☐ Ecological, evolutionary & environmental sciences

For a reference copy of the document with all sections, see nature.com/documents/nr-reporting-summary-flat.pdf

Life sciences study design

All studies must disclose on these points even when the disclosure is negative.

Sample size	No statistical methods were used to predetermine sample size. Three cryo-EM datasets, each consisting of thousands of micrographs were collected for cryo-EM structure determination (4965 micrographs were used in total) The number of micrographs in each data set depended on the ability to make 3D reconstructions and the availability of microscopy time.
Data exclusions	CryoEM data were processed with CryoSPARC 3.1 and low-quality micrographs were excluded for high-resolution 3D reconstruction using common exclusion criteria in cryo-EM data processing, which is part of the data processing pipeline (for example, low estimated CTF or high contamination by ice or ethane). No data were excluded from functional assays and confocal imaging.
Replication	Six replicates on each BRET assay (Supplementary Figure 1, 8, 10, 16 and 21), three independent experiments on GTP turnover assay (Supplementary Figure 29), three replica on molecular dynamics simulations (Supplementary Figure 14 and 23), three independent experiments on ELISA-based surface expression assay (Supplementary Figure 9, 15 and 20), and multiple runs of each Ca2+ signaling assay as described in Supplementary Table 2 were successfully performed. Three fields of view were randomly chosen in each confocal dish using DAPI channel to avoid bias in cell selection (Supplementary Figure 9, 15 and 20) All attempts at replication were successful.
Randomization	Randomization is not relevant to this structural study, because the single particle cryo-EM analysis is based on randomly distributed particle subsets and particles for 3D reconstruction were randomly assigned to calculate gold-standard FSC.
Blinding	Blinding is not relevant to this study as no subjective allocation was involved.

Reporting for specific materials, systems and methods

We require information from authors about some types of materials, experimental systems and methods used in many studies. Here, indicate whether each material, system or method listed is relevant to your study. If you are not sure if a list item applies to your research, read the appropriate section before selecting a response.

Materials & experimental systems

n/a	Involved in the study	
☐	☒	Antibodies
☐	☒	Eukaryotic cell lines
☒	☐	Palaeontology and archaeology
☒	☐	Animals and other organisms
☒	☐	Human research participants
☒	☐	Clinical data
☒	☐	Dual use research of concern

Methods

n/a	Involved in the study	
☒	☐	ChIP-seq
☒	☐	Flow cytometry
☒	☐	MRI-based neuroimaging

Antibodies

Antibodies used	Rabbit anti-FLAG monoclonal antibody (Cell signaling Technology, D6W5B), Goat anti-rabbit IgG polyclonal antibody HRP conjugate (Enzo Life Sciences, ADI-SAB-300-J)
Validation	All antibody were validated by purified target protein in Western blot

Eukaryotic cell lines

Policy information about cell lines

Cell line source(s)	Sf9 insect cells (Expression Systems, 94-001F), HEK293T cells (ATCC, CRL-3216)
Authentication	The Sf9 and HEK293T cell lines were authenticated by the manufacturers and not by our group.

Mycoplasma contamination	The HEK293T cells were not tested for mycoplasma contamination. They were not cultured for prolonged time periods.
Commonly misidentified lines (See ICLAC register)	No commonly misidentified lines were used in this study.
